# Antioxidant Properties and In Vitro Biocompatibility of Monodispersed Cuprous Oxide (Cu_2_O) Nanoparticles

**DOI:** 10.1021/acsomega.6c03242

**Published:** 2026-08-06

**Authors:** Laura Maria Slavu, Maria Elena Giordano, Sanosh Kunjalukkal Padmanabhan, Gayatri Udayan, Giorgio Capuzzelo, Antonio Licciulli, Maria Giulia Lionetto, Rosaria Rinaldi

**Affiliations:** † Department of Mathematics and Physics “Ennio De Giorgi”, 201812 University of Salento, Via Monteroni, 73100 Lecce, Italy; ‡ Department of Biological and Environmental Sciences and Technologies (DiSTeBA), University of Salento, 73100 Lecce, Italy; § Department of Engineering for Innovation, University of Salento, 73100 Lecce, Italy

## Abstract

The increasing prevalence of oxidative stress-related disorders underscores the need for safe and effective nanomaterials with strong antioxidant properties and good biocompatibility. In this study, copper oxide nanoparticles (Cu_2_O NPs) were successfully prepared by a simple chemical method and evaluated for their biocompatibility and antioxidant properties. The prepared NPs were characterized by transmission electron microscopy (TEM), X-ray diffraction (XRD), Fourier transform infrared spectroscopy (FTIR), and dynamic light scattering (DLS). Results showed that Cu_2_O NPs are monodispersed with a spherical shape and an average diameter of 88 ± 6 nm. In vitro biocompatibility studies on 3T3 murine fibroblasts revealed that Cu_2_O NPs exhibited no significant cytotoxic effects at lower concentrations (0.07–7.15 μg/mL), indicating their potential use in biomedical applications within this range. Increasing the concentration from 7.12 μg/mL to 100 μg/mL resulted in a pronounced reduction in cell viability and oxidative stress induction, as assessed using the Almar Blue test and the intracellular CM-H_2_CDFDA probe, respectively. DPPH scavenging and TMB kinetic assays demonstrated significant antioxidant activity (IC_50_ = 7.05 μg/mL) and catalytic properties of Cu_2_O NPs. This study provides a novel and systematic correlation between physicochemical properties, catalytic activity, and biological response, identifying a potential range of biocompatibility for the biomedical application of Cu_2_O nanoparticles.

## Graphical abstract

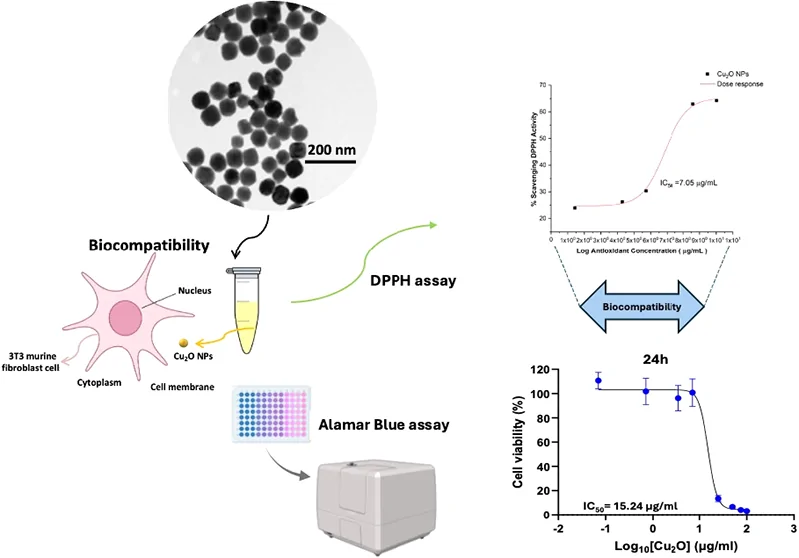

## 1 Introduction

Researchers have consistently shown great interest in metal oxide materials because of their complex and diverse structures, which make them suitable for a wide range of potential applications. When engineered at the nanoscale, these materials exhibit exceptional properties that exceed those of their bulk counterparts.^1^ Copper-based metal oxide materials, in their cupric (CuO) and cuprous (Cu_2_O) forms, because of their transition-metal nature, multivalence, excellent catalytic properties, abundant availability, and cost-effectiveness, are widely used in a variety of applications. Cu_2_O, a p-type semiconductor with a 2.17 eV band gap, is a promising material used in gas sensors, electrochemical sensors, photocatalysis, photosensing, and supercapacitors.^2–4^ Cu_2_O nanoparticles (NPs) have demonstrated strong antimicrobial properties against a wide range of microorganisms, including bacteria, fungi, and viruses. They hold great potential for developing new treatments for infectious diseases, especially in cases where conventional antibiotics are no longer effective due to the emergence of drug-resistant strains.^5^ Despite the promising applications of Cu_2_O NPs in biomedicine, their use for diagnostic and therapeutic purposes raises major concerns due to the toxic effects observed in studies involving various vertebrates (specifically mammalian cells) and invertebrates.^6^ The size, surface charge, and dissolution of nanoparticles are key factors contributing to the *in vitro* and *in vivo* toxicity of NPs.^7^ Therefore, biocompatibility and nontoxicity are crucial criteria for selecting nanoparticles for clinical research. Finally, studies have shown that they may be effective in inhibiting the growth and proliferation of cancer cells while leaving healthy cells unharmed.^8^ In addition to their antimicrobial properties, Cu_2_O nanoparticles exhibit strong antioxidant activity, which can help to reduce oxidative stress and inflammation in the body. This makes them valuable for a variety of health-related applications, including the prevention and treatment of conditions linked to oxidative stress, such as cardiovascular diseases and neurodegenerative disorders.^9^ The antioxidant properties of Cu_2_O NPs depend on their nature, polymorphism, crystal structure, chemical composition, surface charge, particle size, surface-to-volume ratio, surface coating, and dispersion state.^10–12^

Cu_2_O can be synthesized using various methods, including chemical reduction, solvothermal, microwave, and ultrasound techniques. Among these, chemical reduction is one of the most widely used methods. However, the search for new, efficient, and cost-effective synthesis methods remains a significant challenge in nanotechnology.^13^ Recent efforts in shape-controlled synthesis of Cu_2_O nanocrystals have led to various morphologies, including cubes, tubes, octahedrons, polyhedrons, solid and porous spheres, disks, nanowires, and more, as shape influences their structural, physical, and chemical properties.^14^ Cu_2_O nanocrystals with varied morphologies have been synthesized using different capping agents: nano- and microcubes with poly­(ethylene glycol) (PEG, Mw = 600) and cetyltrimethylammonium chloride (CTAB); submicrometer octahedrons with poly­(vinylpyrrolidone) (PVP); nano octahedrons with Triton X-100; and hollow spheres (300–600 nm) with deionized gelatin.^15^ Despite the promising antioxidant and catalytic properties of Cu_2_O nanoparticles, a clear understanding of the relationship among their physicochemical characteristics, redox activity, and biocompatibility remains lacking, particularly in defining a safe and effective concentration range for biomedical applications.

Herein, we report a simple method for the shape-controlled synthesis of cuprous oxide nanoparticles with spherical morphology. This method involves the reduction of CuCl_2_ with ascorbic acid in the presence of poly­(vinylpyrrolidone) (PVP) as a capping agent. Monodispersed spherical Cu_2_O nanoparticles were synthesized, characterized, and evaluated for antioxidant activity using DPPH and catalytic activity using the TMB assay. *In vitro* biocompatibility was assessed by Alamar Blue assay on murine 3T3 fibroblast cells for potential nanomedicine applications. The combined use of murine 3T3 fibroblast cells and the Alamar Blue assay, which assesses metabolically active cells, is a widely used approach in nanomaterials biocompatibility testing.^16^

## 2 Materials and Methods

### 2.1 Synthesis of Cu_2_O NPs

All chemicals and reagents were of analytical grade and used as received without further purification. In this experiment, copper­(II) chloride dihydrate (CuCl_2_·2H_2_O, >99%), sodium hydroxide (NaOH, >99%), l-ascorbic acid (L-AA, C_6_H_8_O_6_, >99%), polyvinylpyrrolidone (PVP MW ∼55,000), 2,2-diphenyl-1-picrylhydrazyl (DPPH, C_18_H_12_N_5_O_6_), and 3,3′,5,5′-tetramethylbenzidine (TMB C_16_H_2_0N_2_.) were purchased from Sigma-Aldrich. Ultrapure water produced with a Smart2Pure 12 system (Thermo Scientific) was used throughout all experiments.

Spherical Cu_2_O NPs were prepared by a typical chemical reduction method with slight modifications that influenced the reaction kinetics and allowed us to obtain quality copper oxide nanoparticles.^17^ In detail, PVP was used as a stabilizing agent and l-ascorbic acid as a reducing agent, while NaOH was used to adjust the pH of the reaction.

Colloidal copper nanoparticles were prepared by dissolving 0.0076 g of CuCl_2_·2H_2_O (0.001 M) and 0.1 g of PVP in 45 mL of water in a 250 mL glass beaker, placed on a hot plate magnetic stirrer set at 25 °C. The resulting solution was left to react for 10 min, followed by sequential injection of 2.0 mL of NaOH solution (0.2 M) and 2.0 mL of ascorbic acid solution (0.15 M) at a flow rate of 5.00 × 10^2^ μL/min. At the end of L-AA addition, a deep orange-colored solution was obtained, which confirmed the formation of copper nanoparticles. Finally, the resulting solution was washed several times with ethanol and water and centrifuged at 11.3*g* rcf for 10 min. Moreover, at the end of each session of centrifugation, the colloidal suspension was left in a bath sonicator at 70 °C for 10 min. This last approach resulted in highly purified nanoparticles. Finally, the obtained nanoparticle pellet was resuspended in 5 mL of water for further characterization. A schematic representation of the synthesis procedure of Cu_2_O NPs is depicted in Figure 1.

**Figure 1:**
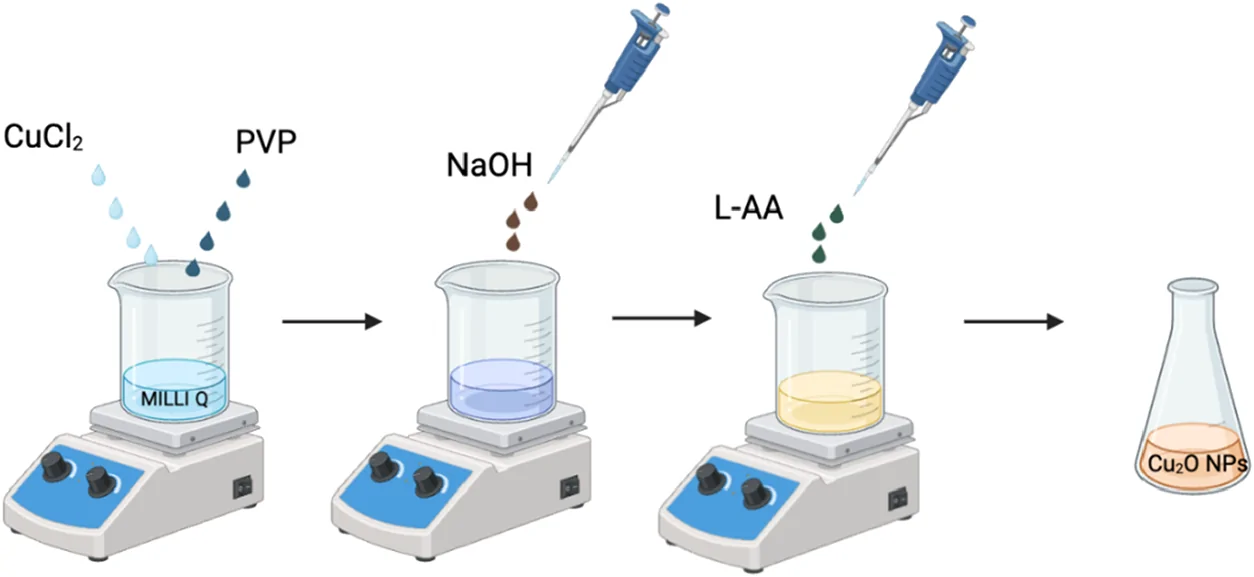
Schematic diagram of the synthesis of Cu_2_O NPs (figure created using BioRender).

### 2.2 Characterization of Cu_2_O Nanoparticles

The morphology of the nanoparticles was analyzed using a transmission electron microscope (TEM) JEOL JEM-1011 operating at an accelerating voltage of 100 kV. The nanoparticles were prepared by dropping a few microliters of nanoparticles onto a Formvar-coated copper grid, just after the completion of the synthesis process. The DLS size distribution and ζ-potential were measured using a Malvern Zetasizer Nano ZSP. All measurements were performed in triplicate. The crystalline structure was analyzed by X-ray diffraction (XRD) using a Rigaku Ultima diffractometer with Cu Kα (λ = 1.5406 Å) radiation operated at 40 kV and 20 mA with a scanning step of 0.02°. For this, 500 μL of nanoparticle suspension was placed on a glass slide and dried at 50 °C overnight. The copper concentration in Cu_2_O dispersion was measured by elemental analysis, using an inductively coupled plasma atomic emission spectrometer (Varian 720 ICP-OES). Before analysis, the samples were digested in a concentrated HCl/HNO_3_ 3:1 (v/v) solution. FTIR (Fourier transform infrared spectroscopy) spectra were acquired using a JASCO FT/IR6800 spectrometer, equipped with a diamond ATR module. The optical properties of the nanoparticles were investigated using a UV–vis spectrophotometer, BioTek Synergy MX Microplate Reader. The absorbance wavelength of the spectrophotometer ranges from 230–999 nm, tunable in 1 nm increments, and has a bandwidth of 2 nm (230–285 nm) and 4 nm (>285 nm). Absorbance spectra were recorded in the range of 350–700 nm.

### 2.3 Antioxidant Activity by Diphenyl-1-PicrylHydrazyl-Hydrate (DPPH) Radical Scavenging Assay

The antioxidant activity of Cu_2_O NPs was investigated by assessing their DPPH scavenging ability, which is a widely used standard method for evaluating antioxidant capacity. The assay was performed by preparing a 0.1 mM ethanolic solution of DPPH with varying concentrations of NPs.

For the analysis, 100 μL of DPPH was mixed with 100 μL of Cu_2_O NPs at different concentrations (1–10 μg/mL) in a 96-well microtiter plate. The solutions were then incubated for 15 min in the dark at room temperature. The absorbance (Abs) of the solutions was recorded at 517 nm using a BioTek Synergy MX multimode multiplate reader. DPPH was used as the negative control and ascorbic acid as the reference. The test was carried out in triplicate. Moreover, to account for the intrinsic absorbance of Cu_2_O NPs at 517 nm, control experiments were conducted by measuring DPPH alone and nanoparticles alone at each tested concentration. The absorbance of the nanoparticles was used as a blank and subtracted from the corresponding sample values before analysis.

The percentage of DPPH radical scavenging activity was calculated using the following equation:

$$\mathrm{DPPH}\;\mathrm{activity}\;\left(\%\right)=[\left(\frac{{\mathrm{Abs}}_{\mathrm{control}}-{\mathrm{Abs}}_{\mathrm{sample}}}{{\mathrm{Abs}}_{\mathrm{control}}}\right)\times 100]$$

where Abs_control_ is the absorbance of DPPH at 517 nm as reference control, Abs_sample_ represents the absorbance of the nanoparticles solution with DPPH at 517 nm, while scavenging % represents the percentage of DPPH reduction (the higher the value, the stronger the antioxidant effect).

Thereafter, the sigmoid dose–response curve was performed to determine the half-maximal inhibitory concentration (IC_50_) by using the following equation:

$$y=A1+\frac{A2-A1}{1+10^{\left({\log x}_{0}-x\right)p}}$$

where *A*1 represents the minimum response value (scavenging % at low NP concentrations), *A*2 represents the maximum response value (scavenging % at high NP concentrations), log *x*_0_ = logarithm of the concentration at which 50% of the effect occurs (IC_50_ in logarithmic scale), *p* = slope of the curve (defines how steep the transition is between the minimum and maximum response), *x* = log of the concentration of the tested substance, and *y* = DPPH scavenging percentage.

### 2.4 Kinetic Data Analysis

The catalytic activity of Cu_2_O NPs was investigated using the theory and methods of enzymatic reaction kinetics, in the presence of H_2_O_2_ (30% w/w) and 3,3′,5,5′-tetramethylbenzidine (TMB) substrate.

The kinetic experiment was designed by varying Cu_2_O nanoparticle (NPs) concentrations under saturating substrate conditions [TMB] = 1 mM, [H_2_O_2_] = 5 M to establish a precise activity–concentration (catalytic dose–response) profile. Maintaining a fixed concentration of TMB and H_2_O_2_ ensures that substrates are not rate-limiting, so any change in reaction rate reflects NPs concentration. While peroxidase-like activity is the primary function, NPs also exhibit antioxidant (catalase-like) activity, and varying NPs concentration allows quantitative assessment of this function per unit mass. The experiment was designed to evaluate the peroxidase-like activity as a function of nanoparticle concentration and represents a dose–response analysis, not a Michaelis–Menten kinetic model. We chose this strategy to establish a direct correlation between the intrinsic catalytic efficiency and the biological response of the nanoparticles to fibroblasts.

We emphasize that the Cu_2_O concentration range adopted for the kinetic assay was selected appropriately to enable comparison with the biologically relevant concentrations investigated in the Alamar Blue, ROS, and DPPH assays, thereby allowing a direct correlation between the catalytic activity and the biological response. As can be observed, nanoparticle concentrations higher than 0.1 mM (15 μg/mL) were not further investigated in the kinetic assay as they are associated with reduced colloidal stability and aggregation phenomena, which could affect both catalytic performance and optical measurements.

For this analysis, NPs solutions at different concentrations were added to a 96-well plate. Subsequently, TMB and H_2_O_2_ were added, and the absorbance of each reaction solution was measured at 652 nm, with measurements taken every 10 s for a total time of 300 s at 35 °C

The concentration of the nanoparticles corresponds to a ≈1.4–12.6 μg/mL range, which was specifically chosen to match the nanoparticle concentrations used in the fibroblast Alamar Blue and ROS assays at low concentration. Under these conditions, the catalytic system operates in a linear regime and does not reach saturation. The variation of the absorbance in time has been calculated from the initial slope of the nanoparticles by plotting the absorbance vs time curve according to the following formula:

$${\mathrm{slope}}_{\mathrm{initial}}=\frac{{\mathrm{Abs}}_{60\;s}-{\mathrm{Abs}}_{0\;s}}{60}$$

Subsequently, the variation of the molar concentration in time *V*_0_ has been calculated from the initial slope by using the Lambert–Beer formula: $V_{0}=\frac{\Delta C}{\Delta t}=\frac{{\mathrm{slope}}_{\mathrm{initial}}}{\epsilon \cdot l}$ , where slope_initial_ is ΔAbs/Δ*t*, ε is the extinction coefficient (39,000 M^–1^ cm^–1^), *l* is the Path length, and *C* is the molar concentration (M).

Thereafter, the catalytic activity was normalized to the mass of nanoparticles and expressed as mass-specific activity according to the formula: $\mathrm{specific}\;\mathrm{activity}=\frac{\mathrm{total}\;\mathrm{activity}\;\left(U\right)}{\mathrm{total}\;\mathrm{mass}\;\left(\mathrm{mg}\right)},\;U/\mathrm{mg}$ , where U represents the amount of material able to convert 1 μmmol of substrate per minute under the experimental conditions. This approach allows us to evaluate the intrinsic catalytic efficiency of the nanoparticles.

In the end, to validate the peroxidase-mimicking activity of Cu_2_O, a typical colorimetric reaction and UV–vis spectrum analysis were performed on all samples used in the kinetic analysis. The absorbance spectrum was recorded in the wavelength range of 400–700 nm.

Initially, the UV–vis spectra were recorded for H_2_O_2_, TMB, and TMB with H_2_O_2_ in the absence of Cu_2_O. Subsequently, UV–vis spectra were recorded for each sample within the entire system Cu_2_O/TMB/H_2_O_2_.

### 2.5 Cell Viability Assessment Using the Alamar Blue Assay

The biocompatibility of the Cu_2_O NPs was assessed in vitro using the Alamar blue assay on mouse embryonic fibroblasts from the NHI/3T3 cell line (ATCC CRL-1658), which are widely used as a cell model for in vitro biocompatibility assessments of new materials. They are employed across various studies to evaluate cell proliferation, morphology, and cytotoxicity, making them a versatile cellular tool.^18–20^ The Alamar blue assay is a proven cell viability test that uses the natural reducing power of live cells to convert resazurin to the fluorescent molecule, resorufin.^21^ This assay is widely employed to evaluate cytotoxic effects, cell proliferation, and metabolic activity in various areas of toxicological research, particularly in the context of safety testing of nanomaterials.^21,22^

NHI/3T3 cells were cultured in Dulbecco′s Modified Eagle Medium (D-MEM) supplemented with 10% (v/v) fetal bovine serum (FBS), 2 mM L-glutamine, and 100 μg/mL penicillin/streptomycin in a humidified atmosphere containing 5% CO_2_ at 37 °C. Cells were diluted to a concentration of 2 × 10^4^ per mL and were seeded in a 96-well plate. They were incubated with Cu_2_O NPs dissolved in complete medium supplemented with 1% (v/v) fetal bovine serum (FBS) at different concentrations ranging from 0.07 μg/mL to 100 μg/mL for 24 and 48 h, respectively. All experiments were conducted between passages 3 and 10 of cell propagation. After treatment with Cu_2_O nanoparticles, Alamar Blue reagent (10 μL) was added to each well and incubated for 2 h under standard culture conditions. Fluorescence (λ_ex_ 560 nm, λ_em_ 590 nm) was measured using Cytation 5 (BioTek Instruments, Winooski, VT), and cell viability was calculated as a percentage vs untreated control cells, after subtraction of the cell-free background. To assess potential interference of Cu_2_O nanoparticles with Alamar Blue fluorescence, cell-free controls containing culture medium, Alamar blue, and Cu_2_O nanoparticles at the same concentrations used in the viability assay were performed and compared with the blank control sample, according to Longhin et al.^21^ No statistically significant difference between the fluorescence values of interference control samples and the fluorescence of blank samples was recorded, indicating lack of interference. The health status of the exposed cells was also assessed by the microscopic observation of cell morphology. Representative images of the cells at the different exposure conditions were acquired in bright-field microscopy using the multimode reader Cytation 5 (40× objective used for visualization).

### 2.6 Intracellular Oxidative Stress Analysis

Intracellular oxidative stress was assessed using the cell-permeant probe CM-H_2_DCFDA (2′,7′-dichlorodihydrofluorescein diacetate) (Thermo Fisher Scientific, Waltham, MA), widely used for detecting reactive oxygen species (ROS) formation and oxidative stress in cells.^23,23,24^ Once inside the cell, intracellular esterases cleave the acetate groups, converting the probe into DCFH, which is retained within the cell; subsequent oxidation by intracellular oxidants converts DCFH to the fluorescent product DCF.

Cells were seeded in 96-well black/clear-bottom plates and allowed to attach for 24 h, then exposed to varying concentrations of Cu_2_O NPs (from 0.07 μg/mL to 100 μg/mL) for 24 h. Then, the cells were washed three times to remove residual Cu_2_O NPs and were charged with 5 μM CM-H_2_DCFDA for 30 min; subsequently, they were washed to remove the extracellular dye. Thereafter, the fluorescence intensity was spectrofluorimetrically measured using the multimode reader Cytation 5. In parallel, to assess potential interference of internalized Cu_2_O nanoparticles with the intracellular probe fluorescence, we included cell-based interference controls consisting of Cu_2_O NP-treated cells but not loaded with CM-H_2_DCFDA. The fluorescence signal measured in these controls, which accounted for potential nanoparticle-related background from internalized particles, was subtracted from the corresponding fluorescence values at each nanoparticle concentration.

Fluorescence values were normalized to the specific cell density (cells/mm^2^) measured for each sample through the bright-field and fluorescence microscopy functions of the multimode reader Cytation 5.

The normalization was performed as follows:

$$\mathrm{normalized}\;\mathrm{fluorescence}=\frac{\mathrm{RFU}}{\mathrm{cell}\;\mathrm{density}}$$

Results were subsequently expressed as fold change relative to control cells (not treated with Cu_2_O NPs), which represented the baseline reference (fold change = 1.0). The normalized fluorescence signal was used as a quantitative marker of the oxidative imbalance. An increase in fold change relative to the control indicates oxidative stress, reflecting a state where ROS accumulation exceeds the antioxidant capacity of 3T3 fibroblasts.

### 2.7 Statistical Analysis

All experiments and measurements were performed in triplicate. For cytotoxicity and oxidative stress data, the statistical significance of data was assessed by one-way ANOVA and Dunnet′s post-test.

## 3 Results

A chemical reduction method was employed for the synthesis, aiming to identify and control the influence of key parameters such as precursor salt concentration, reducing agent concentrations, reaction time, and pH. The first step was to investigate the interaction between CuCl_2_ and the reducing agent l-ascorbic acid while keeping the concentrations of the other chemical reagents (NaOH and PVP) constant. The nanoparticles were characterized by XRD and TEM to determine their crystal structures and morphologies. Based on these data, we observed that the optimal conditions involved a molar ratio of 2:1 between CuCl_2_ and AA (Tables S1 and S2 and Figure S1). A second key parameter considered was the reaction time interval between the addition of NaOH and L-AA. Precise timing was required during the sequential addition of these reagents to prevent premature reduction and nanoparticle aggregation. To investigate this, various time intervals ranging from 1 to 10 min were tested to determine the optimal condition. For this purpose, DLS and TEM analyses were employed to examine the size, dispersion, and morphology of the nanoparticles in the colloidal solutions at different time intervals (Table S3 and Figure S3). In our case, monodispersed nanoparticles resulted at a time interval of 5 min.

The last parameter investigated was pH, which depended on the volume of NaOH (0.2 M) added during synthesis. Different volumes (2.0–3.5 mL) were tested to ensure an alkaline environment. Using 2.0 mL of NaOH resulted in a pH of ∼7.4, which favored the formation of stable Cu_2_O nanoparticles (Table S4). Under these conditions, the solution showed a characteristic color change from blue to green to reddish-orange, confirming the progressive formation of Cu_2_O NPs.

### 3.1 TEM Analysis

The morphology and particle size distribution of the nanoparticles were examined by TEM analysis. The results from characterization indicate that the nanoparticles are monodispersed with a regular size and a quasi-spherical shape. The size of the as-synthesized NPs was in the range of 65–100 nm with a mean of 88 ± 6 nm (Figure 2), measured by ImageJ software (measuring *n* > 100 nanoparticles from multiple TEM micrographs).

**Figure 2:**
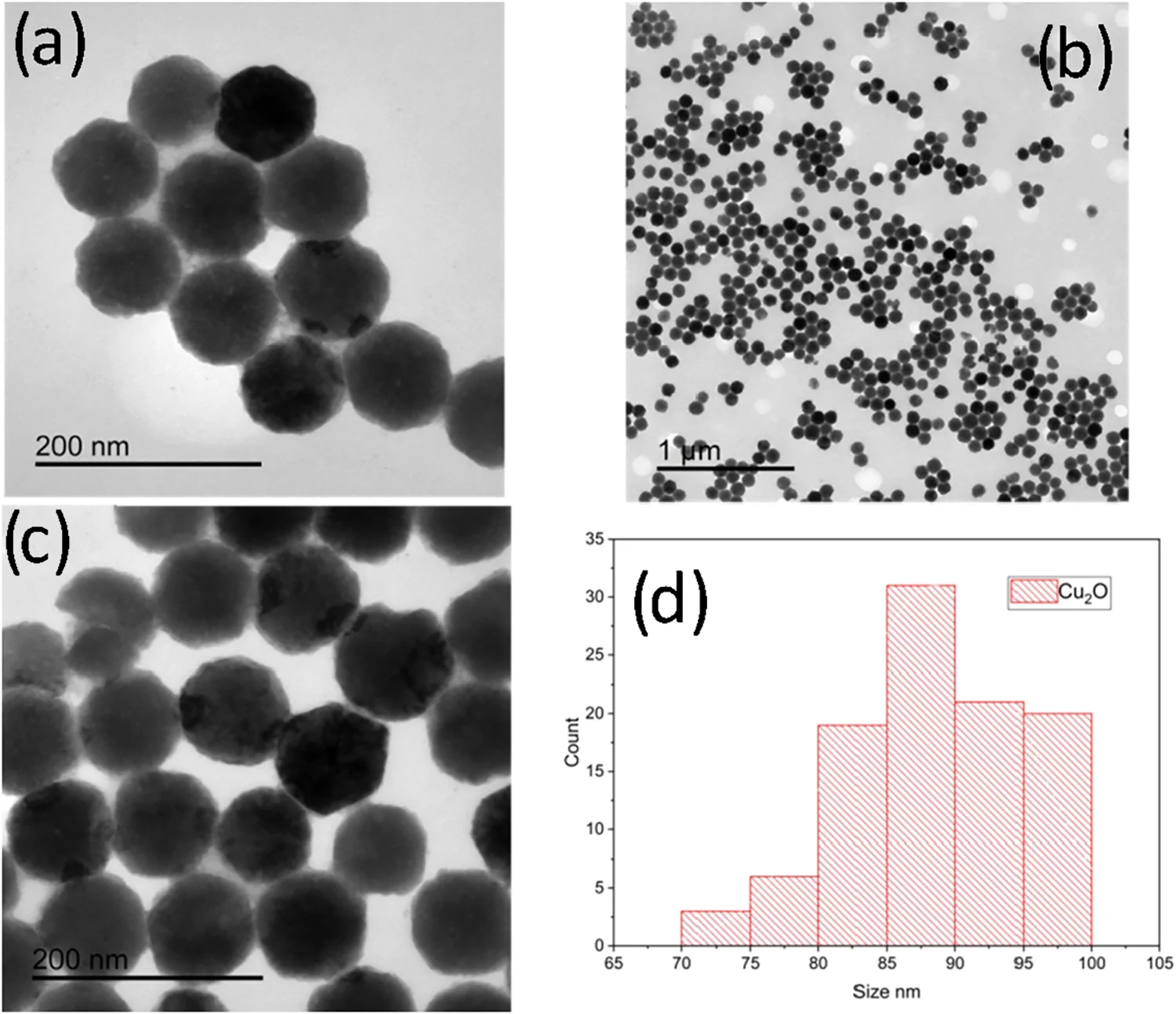
(a–c) TEM micrographs at different magnifications and (d) particle size distribution of Cu_2_O NPs measured by ImageJ software.

### 3.2 DLS Analysis

The dynamic light scattering (DLS) technique provides valuable insights into the hydrodynamic size distribution and colloidal stability of nanoparticles. To assess their physical stability over time, DLS measurements were conducted at two time points: immediately after synthesis (*T* = 0) and after 6 months of storage at room temperature (*T* = 6). This analysis aimed to evaluate potential changes in nanoparticle dispersion and aggregation over time, ensuring the stability of the colloidal system. Notably, the analysis revealed a negligible change in polydispersity index (PDI), with values of 0.107 and 0.129, and mean sizes between ∼152 and 170 nm, respectively (Figure 3a,b). Moreover, the observed PDI < 0.2 clearly indicates the narrow size distribution of the nanoparticles.^25^ The electrostatic stability of copper oxide (Cu_2_O) nanoparticles was assessed by measuring their ζ-potential, resulting in a value of −14 mV (Figure 3c). This value indicates moderate colloidal stability, characterized by a net negative surface charge that promotes electrostatic repulsion between the particles, sufficient to prevent immediate aggregation. However, combined with the hydrodynamic diameter and the good polydispersity index (PDI 0.107), the recorded ζ-potential confirms the formation of a homogeneous and well-dispersed nanoscale system.

**Figure 3:**
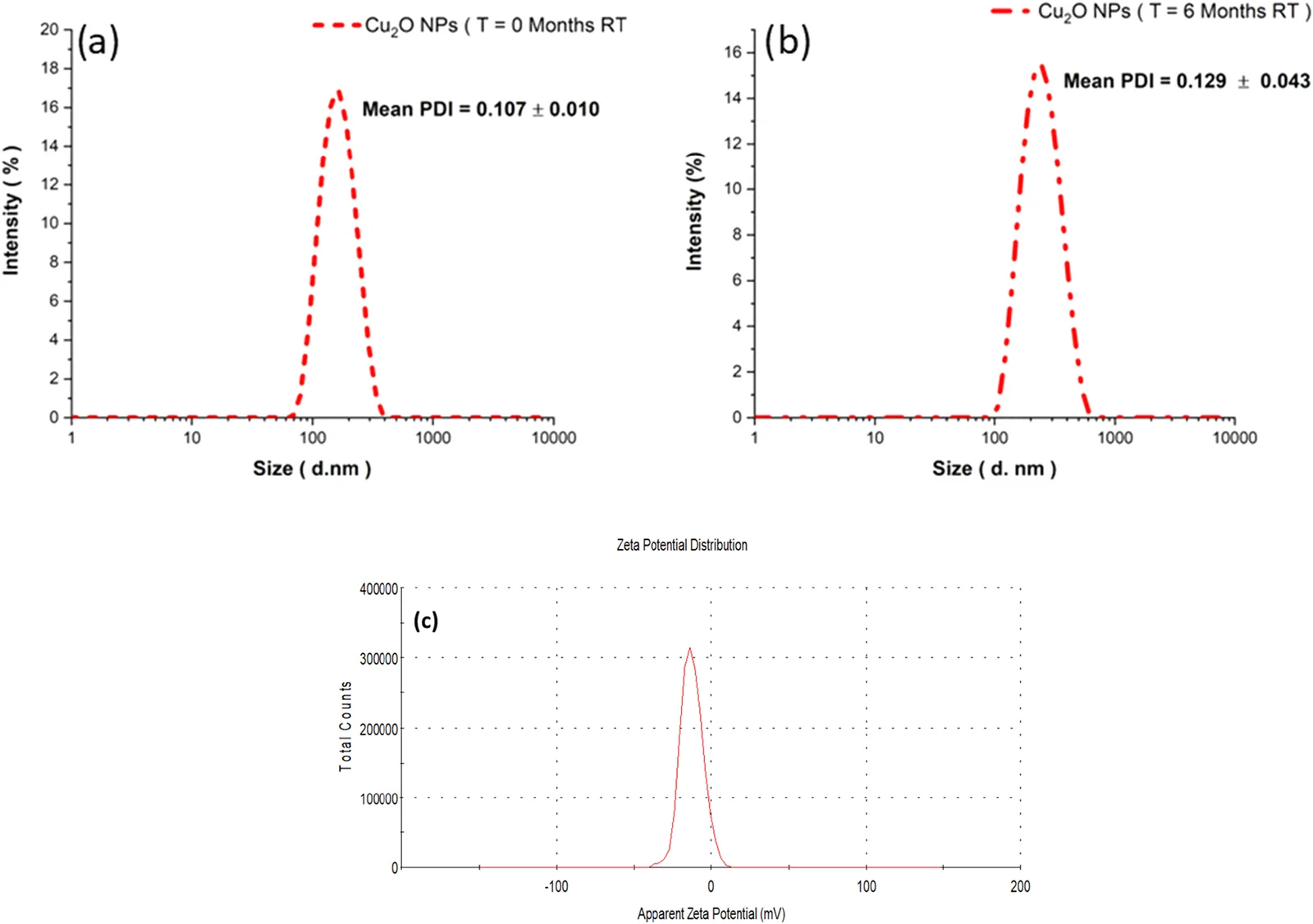
DLS analysis (a) after synthesis and (b) after 6 months and (c) ζ-potential of measurement data for Cu_2_O NPs.

### 3.3 XRD Analysis

X-ray diffraction was used to determine the crystal structure of Cu_2_O. As shown in Figure 4, the marked peaks with 2θ values of 29.5°, 36.4°, 42.3°, 61.4°, 73.5°, and 77.4° correspond to (110), (111), (300), (221), (311), and (222) planes of the crystalline Cu_2_O, respectively, and all of the diffraction peaks are well consistent with the Cu_2_O crystal structures (JCPDS 03-0898).^26^ All of the diffraction peaks follow space group *Pn*3̅*m*, which is a BCC cubic phase of copper nanoparticles, indicating the successful synthesis of cuprous oxide. Moreover, the intense peaks at (111) indicate a highly crystalline nature of the nanoparticles. Using the integral breadth of the XRD peak and applying the Debye–Scherrer equation, the crystallite size was calculated to be 61.5 nm.

**Figure 4:**
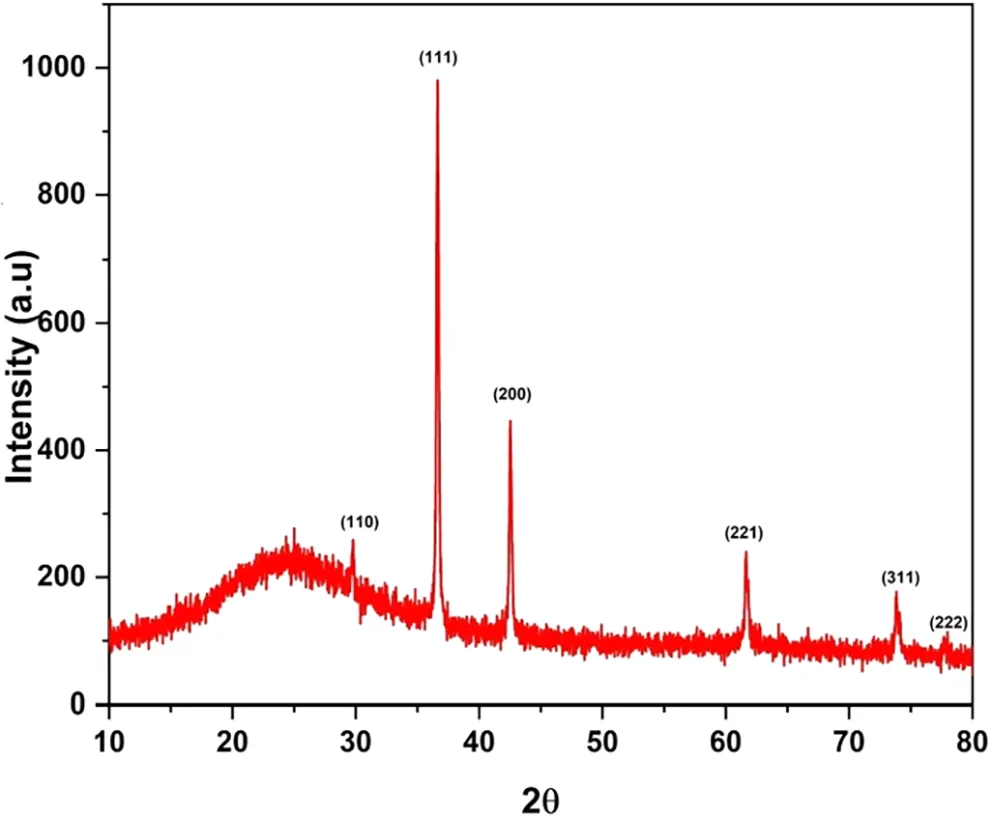
XRD plots of Cu_2_O NPs.

### 3.4 FTIR Analysis

FTIR analysis reveals the successful stabilization of Cu_2_O nanoparticles by poly­(vinylpyrrolidone) (PVP), as indicated by the characteristic bands corresponding to C=O (∼1650 cm^–1^), C–N (∼1300 cm^–1^), and C–H (∼2900 cm^–1^) stretching vibrations.^27^ Importantly, the appearance of a strong absorption band in the low-frequency region (∼500–600 cm^–1^) confirms the formation of the Cu–O bond within the Cu_2_O crystal lattice. On the other hand, a slight shift in the C=O stretching frequency relative to pure PVP suggests a strong correlation between the pyrrolidone ring and the copper surface atoms, thereby preventing nanoparticle aggregation. Notably, the absence of distinct peaks associated with ascorbic acid (AA), such as strong C=O or O–H signals from carboxyl and hydroxyl groups, suggests that AA is no longer present on the nanoparticle surface (Figure 5).^28^

**Figure 5:**
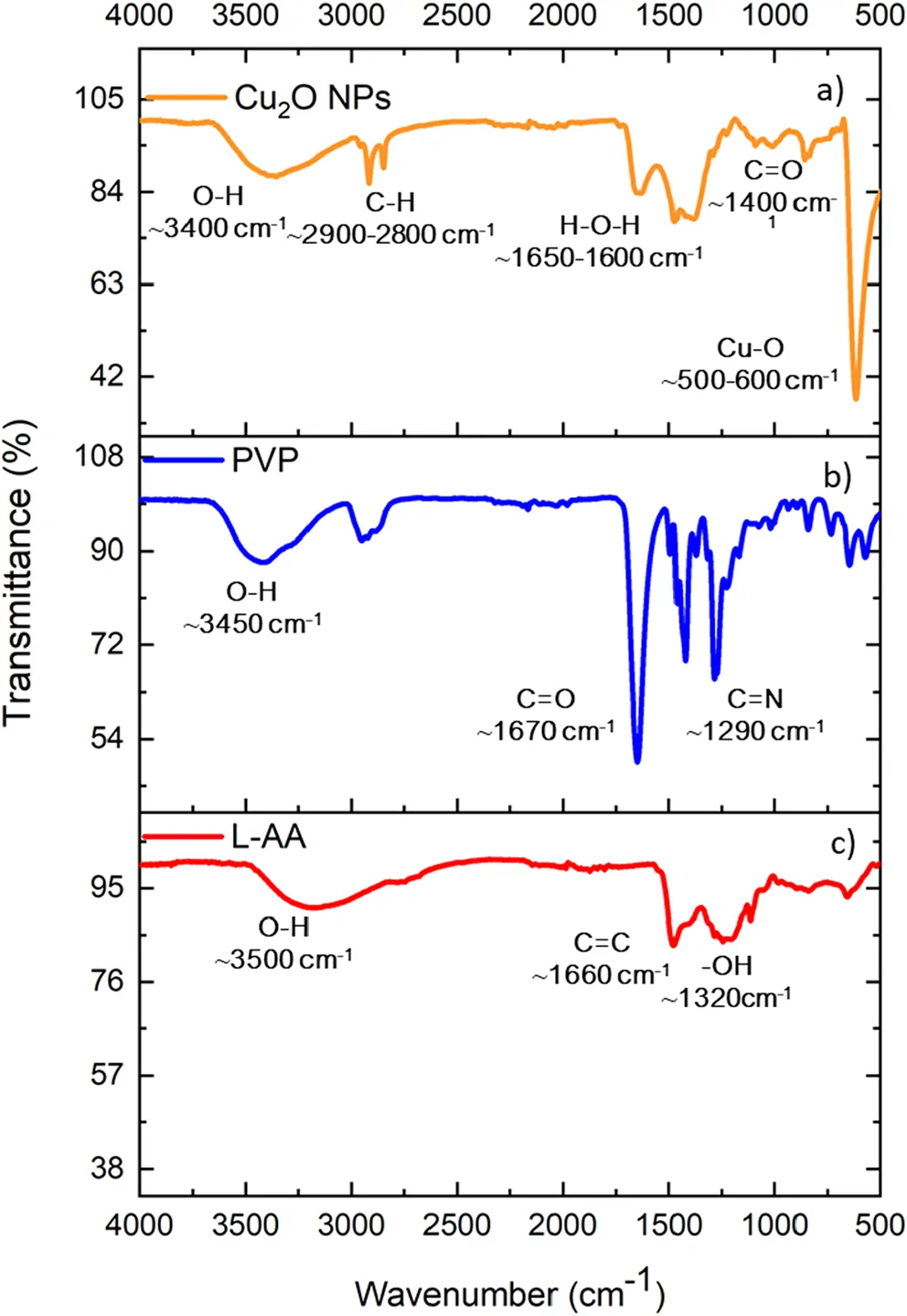
FTIR Spectrum of (a) Cu_2_O nanoparticles, (b) pure PVP, and (c) L-AA.

### 3.5 UV–Vis Analysis

The optical properties of the synthesized Cu_2_O nanoparticles were recorded using UV–vis spectroscopy in the wavelength range of 280–700 nm (Figure 6). From the analysis, a strong absorption peak appears at around 450 nm, which confirms the characteristics of cuprous oxide and is consistent with previously reported spectra.^29^ In addition to the SPR peak at 450 nm, a continuous rise in extinction is observed below 350 nm. This trend is attributable to interband electronic transitions (from the valence band to the conduction band), an intrinsic feature of the semiconductor nature. This further supports the phase purity of Cu_2_O, in agreement with the XRD data.

**Figure 6:**
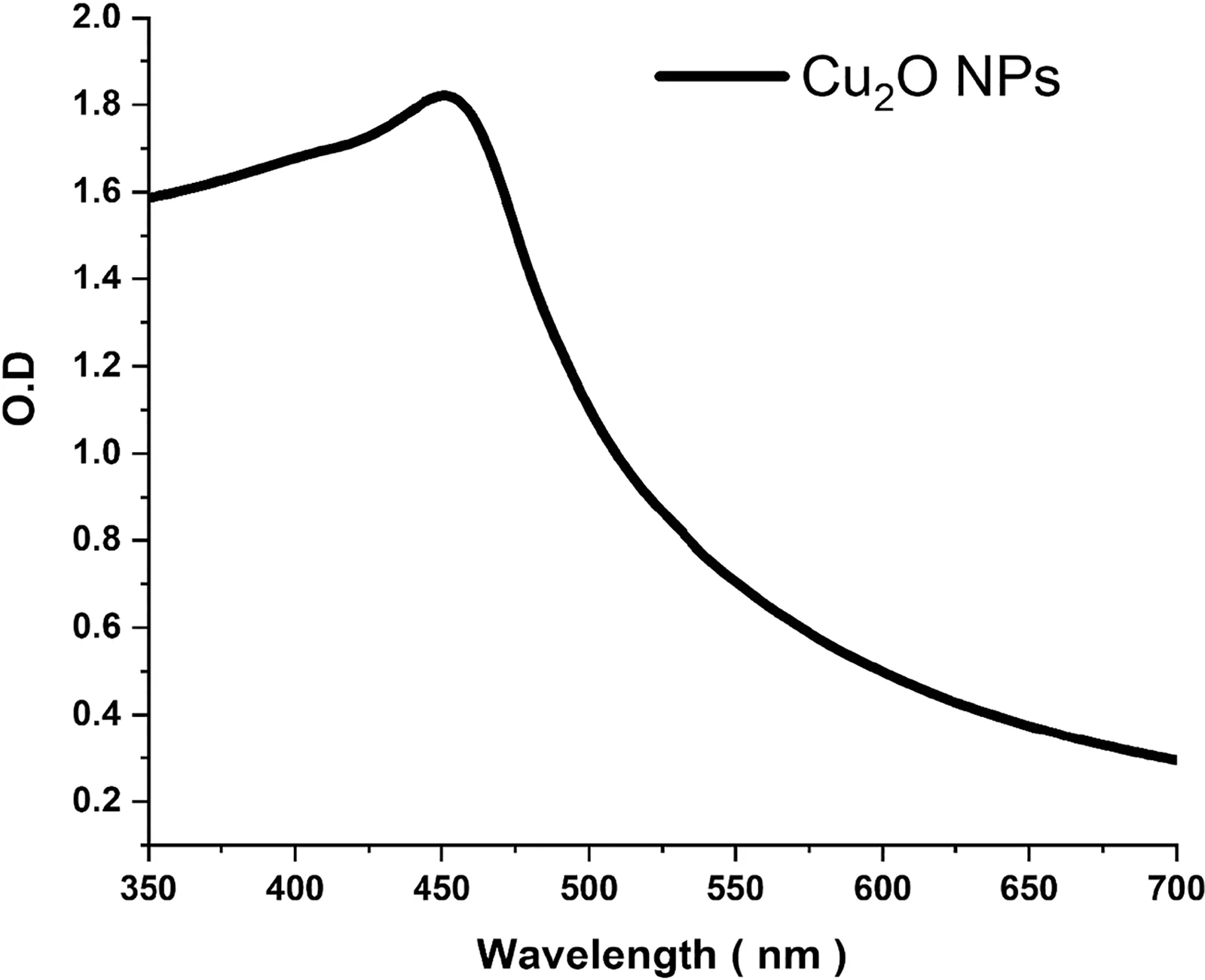
UV–visible absorption spectra of Cu_2_O NPs synthesized.

### 3.6 Radical Scavenging Assay DPPH (2,2-Diphenyl-1-Picrylhydrazyl)

Cu_2_O nanoparticles displayed a dose-dependent behavior in the DPPH radical scavenging assay. This result is in accordance with previous literature reports.^30–32^ After correction for the intrinsic absorbance of the nanoparticles (see the Supporting Information for control experiments with DPPH alone and Cu_2_O NPs alone Figure S5), a clear antioxidant effect was observed at low concentrations, with scavenging activity increasing from 21.96% at 1 μg/mL to 64.3% at 10 μg/mL, corresponding to an IC_50_ of 7.05 μg/mL, indicating relatively high radical scavenging efficiency. In contrast, at higher concentrations, it declined progressively from 48.4% to 34.9% and had an IC_50_ of 59.3 μg/mL (Table 1 and Figures 7a,b and 8).

**Figure 7:**
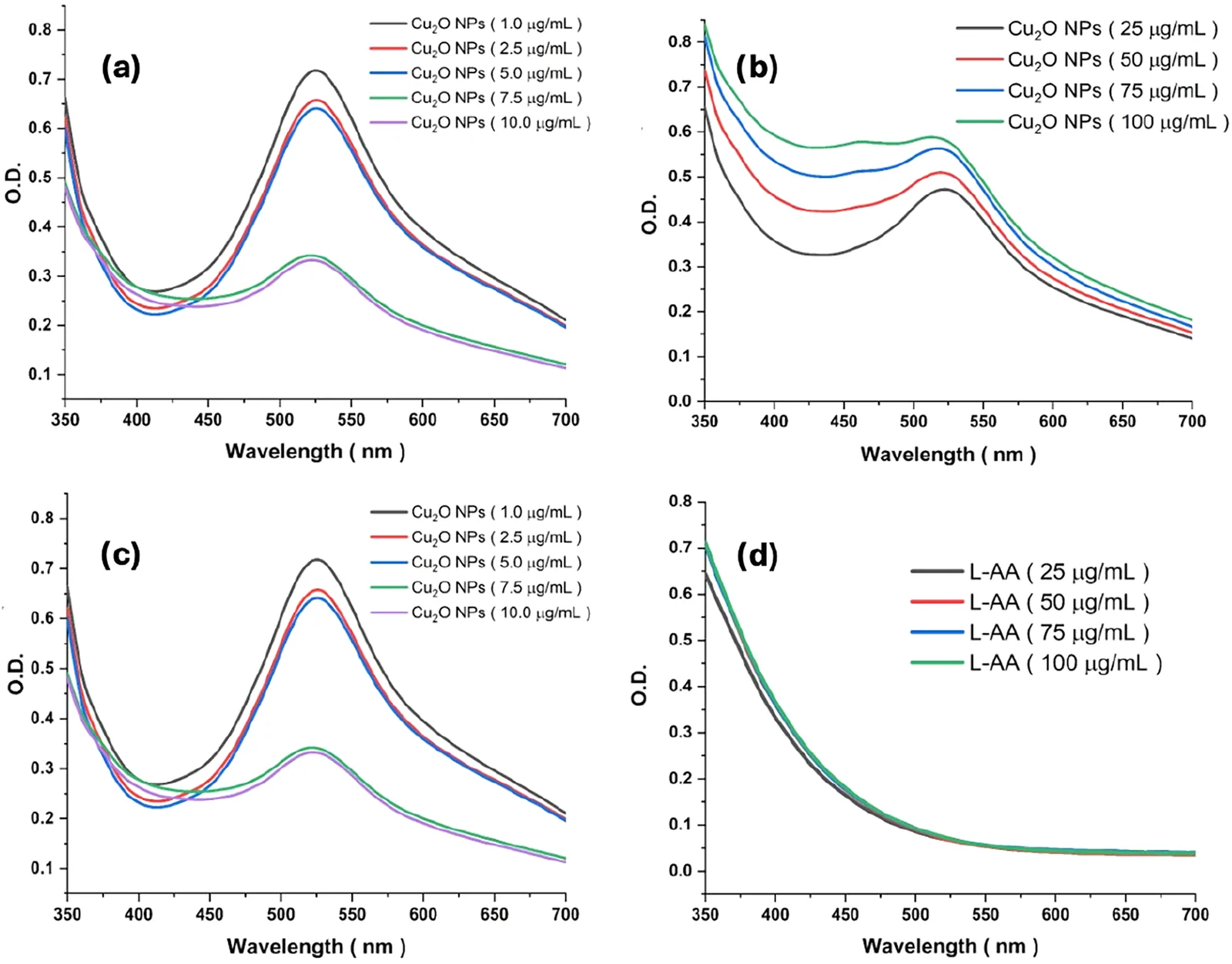
UV–vis absorption spectra (350–700 nm) of DPPH in the presence of Cu_2_O NPs at different concentrations and the ascorbic acid control. Panels (a) and (b) show the behavior of Cu_2_O NPs at low (1–10 μg/mL) and high (25–100 μg/mL) concentrations, respectively, while panels (c) and (d) report the comparison with ascorbic acid (AA) in the same concentration ranges. The spectral variations, in particular at the characteristic wavelength of DPPH (∼517 nm), reflect the free radical scavenging capacity and allow the comparison between the antioxidant activity of the nanoparticles and that of the positive control.

**Figure 8:**
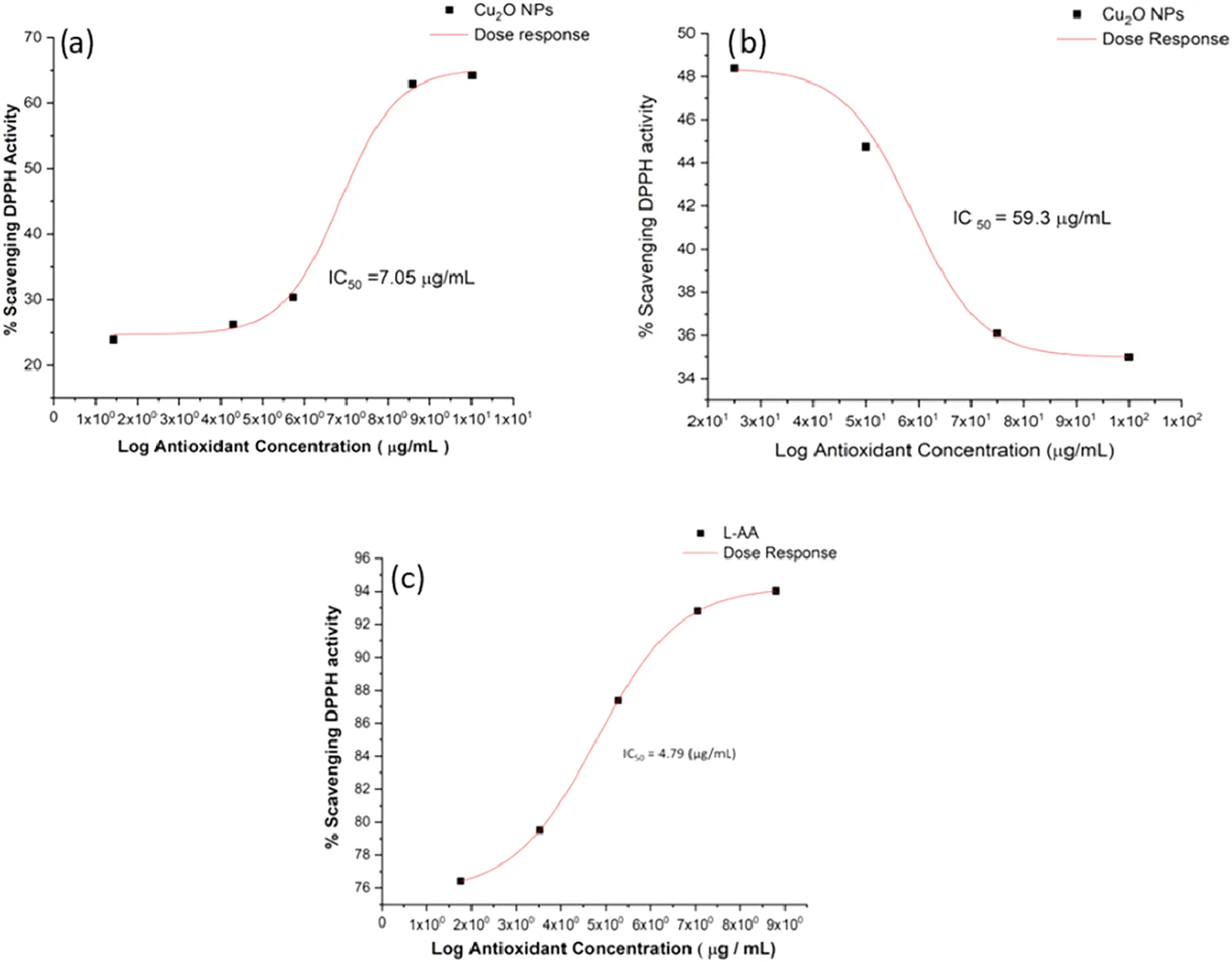
DPPH radical scavenging activity of Cu_2_O NPs and l-ascorbic acid (L-AA). (a) Antioxidant activity of Cu_2_O at low to medium concentrations, showing a dose-dependent increase in scavenging capacity with an IC_50_ = 7.05 μg/mL. (b) Scavenging profile at high concentrations of Cu_2_O NPs, where a significant decrease in activity is observed (IC_50_ = 59.3 μg/mL for the loss of activity), confirming the transition from antioxidant to a pro-oxidant state. (c) Dose–response curve as a standard positive control of L-AA (IC_50_ = 4.79 μg/mL). All of these results are in agreement with the Alamar Blue and ROS assays, identifying the concentration of 7.05 as the critical threshold for the nanoparticles’ functional inversion.

**Table 1:** Antioxidant Activity of Cu_2_O NPs and AA

compound degradation % of Cu_2_O and L-AA by varying concentration (μg/mL)					
Cu_2_O NPs	1 μg/mL	2.5 μg/mL	5 μg/mL	7.5 μg/mL	10 μg/mL
low concentration L-AA	21.92	29.23	30.4	62.9	64.23
low concentration Cu_2_O NPs	68.21	76.52	79.73	83.61	90.25
	25 μg/mL	50 μg/mL	75 μg/mL	100 μg/mL	
high concentration	48.39	43.74	38.09	34.88	

This inverted sigmoidal pattern indicates a transition from antioxidant to pro-oxidant behavior at elevated nanoparticle concentrations. Such dual activity is consistent with the redox cycling properties of copper (Cu^+^/Cu^2+^), which can donate electrons at low doses but may promote reactive oxygen species (ROS) generation and oxidative stress at higher concentrations.^26,27^

On the other hand, ascorbic acid, which is a widely recognized antioxidant, confirmed its potent scavenging activity with values increasing from 68.21% at 1 μg/mL to 90.25% at 10 μg/mL. Moreover, it has reached an IC_50_ plateau at concentrations above 5 μg/mL, reflecting its well-known potency as a direct radical scavenger (Table 1 and Figures 7c,d and 8a–c).^32^

Despite the dual behavior of Cu_2_O compared to AA, its antioxidant activity remains significant, given that the FTIR analysis confirmed the absence of residual AA on the nanoparticle surface.

The data confirm that the antioxidant activity is intrinsic to Cu_2_O NPs and is not due to residual l-ascorbic acid. FTIR analysis verified the absence of surface-bound AA, indicating that radical scavenging arises from the surface redox processes. This supports a nanozyme-like mechanism driven by Cu^+^/Cu^2+^ redox cycling and interfacial electron transfer.

### 3.7 Kinetic Analysis

First, the absorbance vs time was used to determine the initial reaction velocity *V*_0_ by calculating the slope at the initial point of each reaction through first derivative analysis. The analysis was performed using nanoparticle concentrations ranging from 0.01 to 0.09 mM, corresponding to the same concentrations (μg/mL) tested in the cell viability assays. The analysis revealed well-defined curves, with the initial linear phase showing a rapid increase over time (Figure 9a). Then *V*_0_ was calculated, where a very clear linear correlation between the concentration of nanoparticles and the initial velocity *V*_0_ is observed (Figure 9b). As seen from the graph, *V*_0_ increases continuously up to 0.241 μmol/min, while a slight decrease is observed at 0.223 μmol/min.

**Figure 9:**
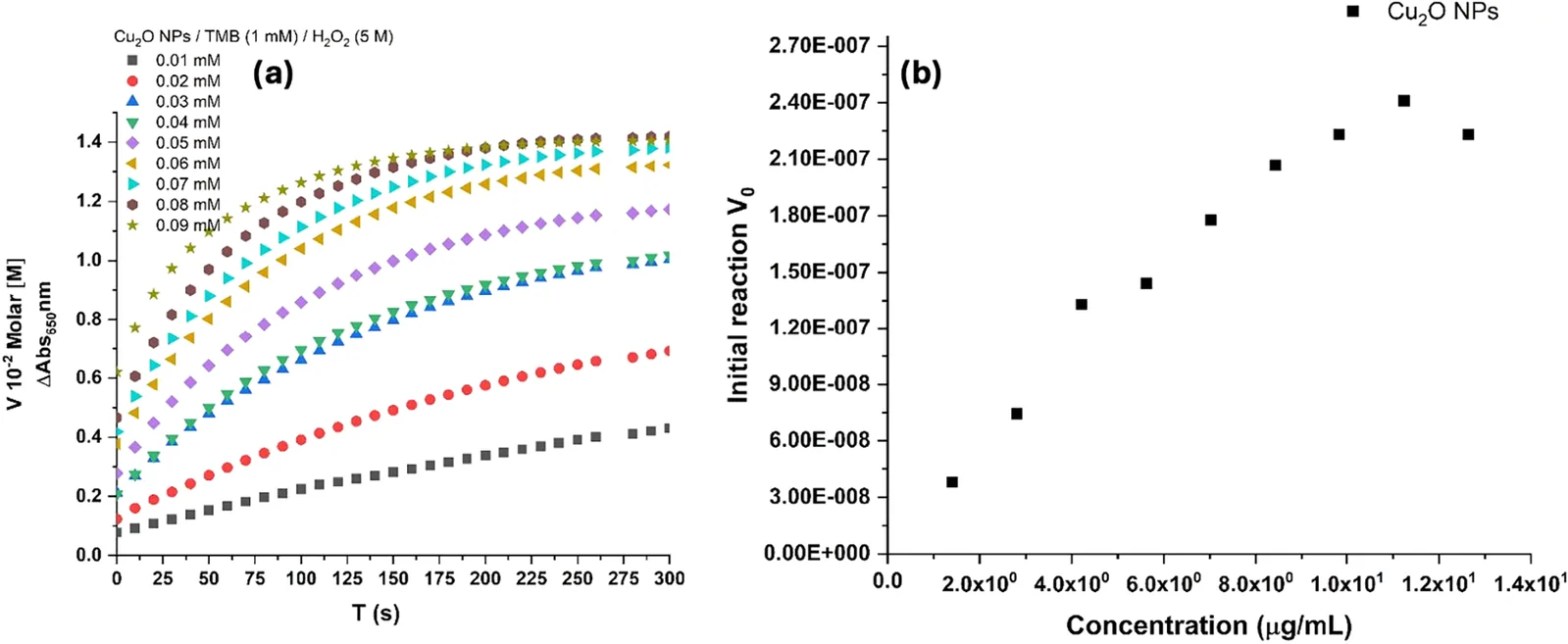
(a) Absorbance changes over time for the nanoparticles and (b) the corresponding initial reaction velocity (*V*_0_) plotted as a function of nanoparticle concentration.

Regarding the specific activity mass, from our data analysis, we observed that the mass-specific activity showed an overall decreasing trend with increasing nanoparticle concentration, from a maximum value of 1.89 U/mg to 1.05 U/mg, with an average of 1.48 ± 0.18 U/mg. Practically, these values indicate that after reaching the peak activity at 1.89, a continuous and progressive decreasing take place up to 1.05 at the highest concentration tested (Table 2 and Figure 10).

**Figure 10:**
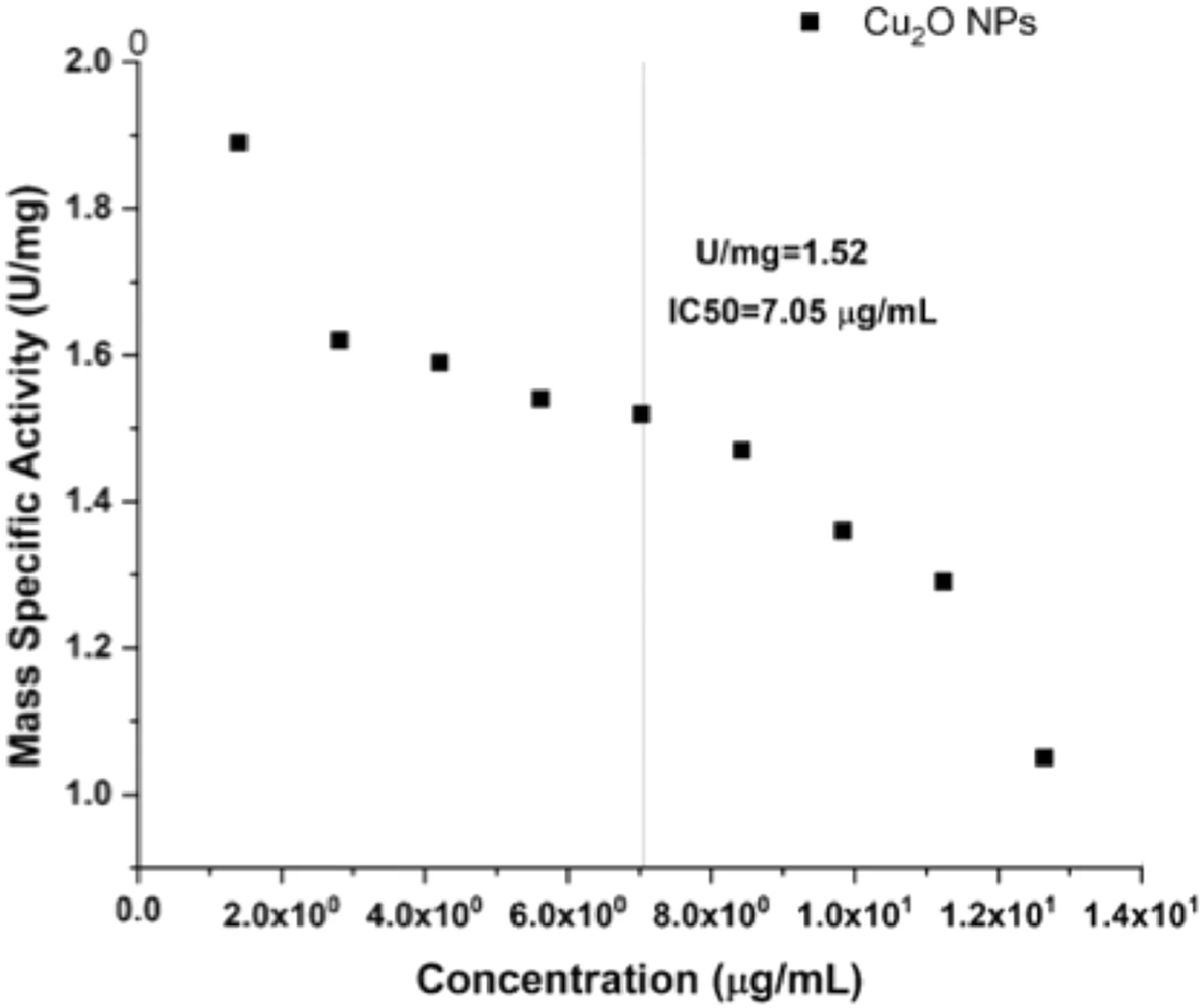
Graphical representation of the dose-dependent decrease in specific activity. The IC_50_ value of 7.05 μg/mL identifies the critical threshold for catalytic efficiency. Below this concentration, the nanoparticles exhibit high specific activity and antioxidant behavior. Conversely, concentrations above this threshold correlate with the transition toward a pro-oxidant profile and the induction of dose-dependent oxidative stress, as confirmed by Alamar Blue, ROS, and DPPH assays.

**Table 2:** Correlations between NPs Concentration (μg/mL), Initial Velocity (*V*_0_ μmol/min), and Mass-Specific Activity (U/mg).

concentration of NPs (μg/mL)	*V*_0_ (μmol/min)	mass-specific activity (U/mg)
1.4	0.038	1.89
2.8	0.074	1.62
4.2	0.132	1.58
5.6	0.144	1.53
7.02	0.177	1.51
8.4	0.206	1.47
9.8	0.223	1.36
11.2	0.241	1.28
12.6	0.223	1.05

This phenomenon is quite common and can be explained as follows: at low concentrations, our nanoparticles present a good catalytic activity, and we are in a regime where the catalytic surface is not saturated, and there are no significant phenomena of self-inhibition or aggregation that limit the reaction. On the other hand, as we expected, a reduction in catalytic efficiency was observed at higher concentrations, which is due to the formation of nanoparticle aggregation on the active sites. These results also explain the scavenging effect observed in the DPPH assay and the resulting protective effect on fibroblasts. Overall, the results indicate that the system operates under conditions close to the ideal regime at low and intermediate nanoparticle concentrations, while at higher catalytic loadings, specific efficiency-limiting effects emerge. Beyond this threshold (e.g., at 25–100 μg/mL), the cumulative catalytic output becomes excessive, leading to the observed transition from an antioxidant effect to a pro-oxidant one.

The data are relevant and demonstrate that the Cu_2_O NPs effectively possess an intrinsic antioxidant activity, quantitatively described by a mass-specific activity. Moreover, this experimental approach supports the application as redox-active and biocompatible nanoparticles in biomedical applications and that their toxicity at higher doses is a direct consequence of an overload of cellular redox activity, not of a change in the chemical properties of the particles. Therefore, by comparing the mass-specific activity with the cytotoxicity data, the IC_50_ of 7.05 μg/mL corresponds to a mass-specific activity of 1.52 U/mg. This value matches perfectly within the safe biological range of catalytic activity, maintaining intrinsic antioxidant activity without inducing cytotoxicity in murine fibroblasts.

### 3.8 Cell Viability Assay and Intracellular Oxidative Stress Assessment

The biocompatibility of the Cu_2_O NP was assessed *in vitro* against 3T3 murine fibroblasts exposed for 24 h to a wide concentration range from 0.07 μg/mL to 100 μg/mL using the Alamar blue assay. As reported in Figure 11a, at the lower concentrations tested from 0.07 μg/mL to 7.15 μg/mL, the cell viability was always close to the control value and, in any case, never fell below 90%. No significant differences (*p* > 0.05, one-way ANOVA and Dunnett′s post-hoc test) were observed with respect to control cells. On the other hand, an increase in concentration from above 7.15 μg/mL up to 100 μg/mL resulted in a strong reduction in cell viability, as shown by the logistic curve. The calculated IC_50_ value corresponded to 15.24 μg/mL. This value obtained for 3T3 fibroblasts is consistent with literature data, as reported values for Cu_2_O-based nanoparticles typically fall within the 20–65 μg/mL range, depending significantly on particle size, surface properties, and the specific biological model used.^33,34^ The health status of the exposed cells was also assessed by the microscopic observation of the cells, as shown in Figure 11b, reporting the appearance of 3T3 murine fibroblasts in control and treated conditions after 24 h exposure to Cu_2_O NP in the whole concentration range from 0.07 μg/mL to 100 μg/mL. It is known that alterations in the size and shape of 3T3 fibroblasts, such as changes in the actin cytoskeleton and cell morphology, can serve as indicators of cytotoxicity.^35,36^ These morphological changes are often accompanied by other signs of cytotoxic stress, such as decreased cell viability.^37^ In our case, no alteration in cell morphology or dimension was detected in the low concentration range tested from 0.07 μg/mL to 7.15 μg/mL, while a further increase of the concentrations up to 100 μg/mL induced alterations in cell morphology such as cell rounding and loss of the typical cell shape, confirming metabolic data provided by the Alamar Blue test.

**Figure 11:**
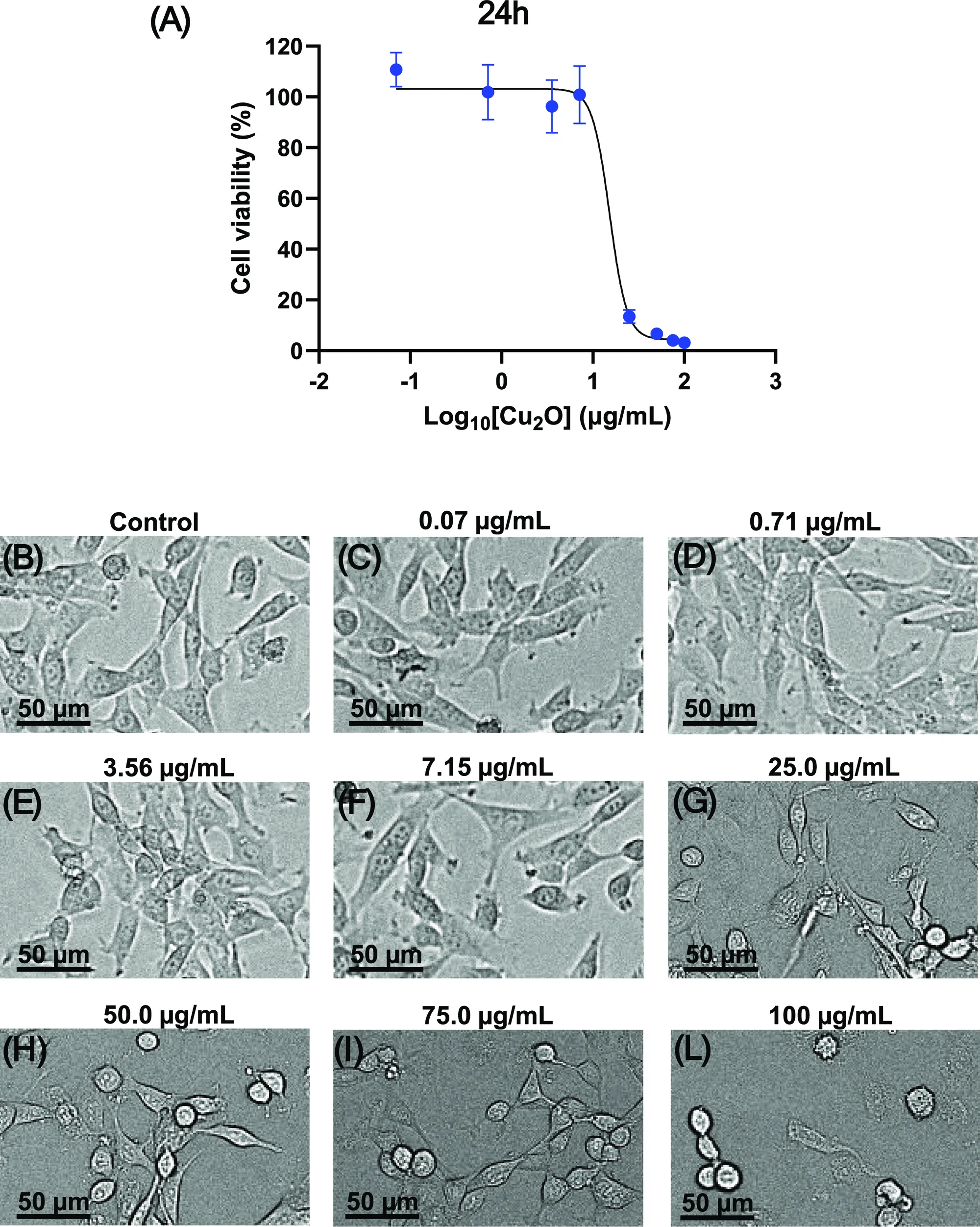
(A) Dose–response curve, expressed as a semilogarithmic scale plot, of the relative viability of 3T3 cells assessed by Alamar Blue assay after 24 h exposure to Cu_2_O NPs. The relative cell viability was referred to control cells (cells not exposed). The Cu_2_O concentrations tested ranged from 0.07 μg/mL to 100 μg/mL. Data were fitted by a four-parameter logistic (4PL) equation with *x* representing the log of concentration. The function was used to calculate the IC_50_ value. Data are expressed as mean ± SD of three independent experiments. (B–L) Representative images of control cells and cells exposed to increasing concentrations of Cu_2_O; the cells were visualized under bright-field microscopy using the multimode reader Cytation 5 (40× objective used for visualization).

To further assess cell viability in response to low concentrations ranging from 0.07 to 7.15 μg/mL and to determine whether the absence of toxicity observed at these levels persists with extended exposure, we treated the cells for 48 h within this concentration range. The results, presented in Figure 12, align with those observed after 24 h at low doses, showing no significant differences compared to the control group. These findings support the in vitro biocompatibility of the tested material across the low concentration range of 0.07–7.15 μg/mL.

**Figure 12:**
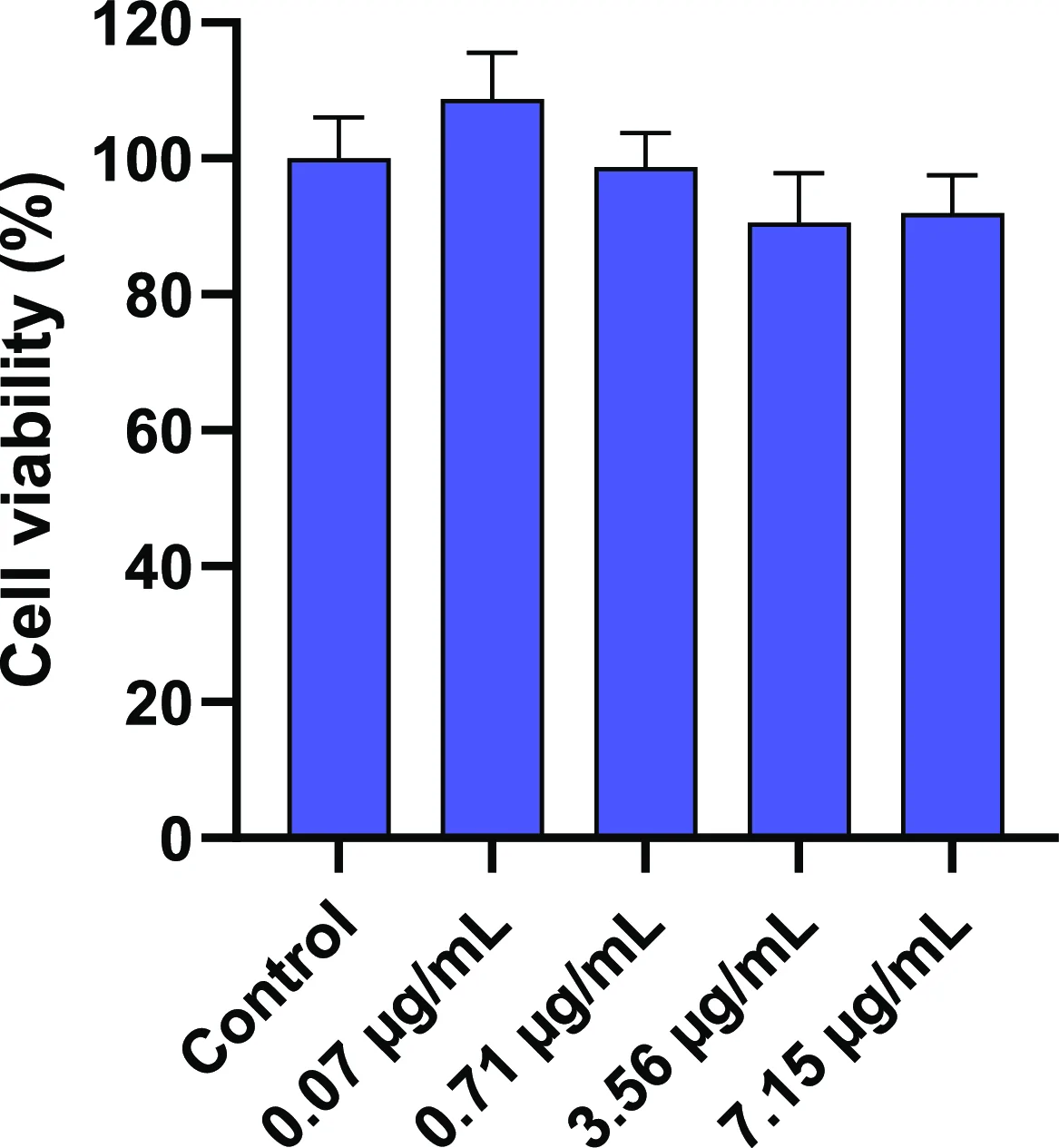
Relative cell viability (assessed by Alamar blue assay) of 3T3 cells exposed for 48 h to Cu_2_O NPs within the following low concentration range 0.07 μg/mL to 7.15 μg/mL. Data are expressed as mean ± SD of three independent experiments.

A comparison between the DPPH radical scavenging assay and the cytotoxicity data shows a clear dose-dependent correlation. Low concentrations of Cu_2_O nanoparticles (≤7–10 μg/mL) exhibited significant antioxidant activity and maintained high 3T3 cell viability, whereas higher concentrations resulted in decreased radical scavenging efficiency and concomitant cytotoxic effects, indicating a transition from antioxidant to pro-oxidant behavior consistent with reduced cell viability. To further investigate this transition at higher concentrations, the impact of Cu_2_O nanoparticles on 3T3 cells in terms of oxidative stress induction was assessed using the ROS-sensitive fluorescent probe CM-H_2_DCFH-DA. Cells were exposed to various concentrations of Cu_2_O nanoparticles (ranging from 0.07 μg/mLto 100 μg/mL) for 24 h. Following exposure, cells were loaded with the fluorescent probe, and the resulting fluorescence was measured. The results, reported in Figure 13, are expressed as the fold change of probe fluorescence normalized to cell density relative to untreated control cells (not exposed to Cu_2_O and charged with the probe). Interestingly, at low concentrations (up to 7.15 μg/mL), no induction of oxidative stress was detected; in addition, treatments with 3.56 μg/mL and 7.15 μg/mL resulted in a significant (*p* < 0.05, one-way ANOVA and Dunnett′s post-hoc test) reduction of probe fluorescence by approximately 15% compared to the untreated control. This decrease could be attributable to an antioxidant action against basal endogenous ROS normally produced during normal cellular metabolism. However, this trend was inverted at higher concentrations starting from 25 μg/mL. At this concentration, the normalized fluorescence increased to a fold change of approximately 1.4× relative to the control. The most marked increase in oxidative stress occurred at the highest concentrations tested of 75 μg/mL and 100 μg/mL, with fold change values exceeding 3.4× relative to the untreated control. Overall, the results obtained with the fluorescent probe correlate with the cell viability data and microscopic observations, confirming the biocompatibility of Cu_2_O NPs within the 0.07–7.15 μg/mL range, while revealing the induction of oxidative stress at higher concentrations.

**Figure 13:**
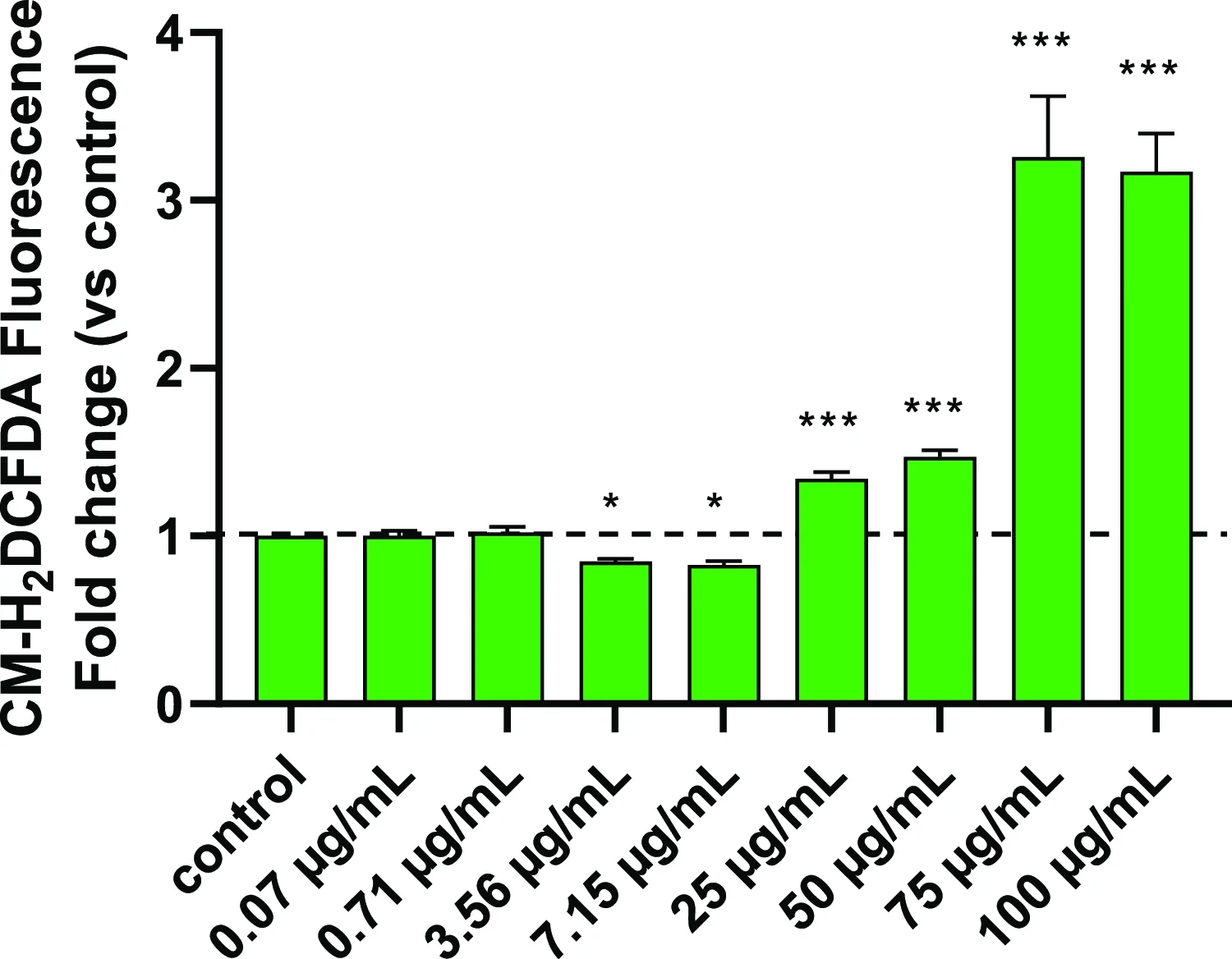
Assessment of intracellular oxidative stress. Intracellular oxidative stress was measured using the ROS-sensitive CM-H_2_DCFH-DA fluorescent probe in 3T3 cells after 24 h of exposure to varying concentrations of Cu_2_O (0.07–100 μg/mL). Data are expressed as the fold change of probe fluorescence normalized to cell density relative to untreated control cells (not exposed to Cu_2_O and charged with the probe, dashed line at *y* = 1). Data are expressed as mean ± SD of three independent experiments. *P < 0.05; ***P < 0.01 (one-way ANOVA and Dunnet′s post-hoc test).

## 4 Discussion

This study aimed to synthesize monodisperse Cu_2_O nanoparticles (NPs) and evaluate their biocompatibility to assess their suitability for safe biological applications. The synthesis conditions were optimized to produce spherical Cu_2_O nanoparticles with a narrow size distribution. The concentration of ascorbic acid played a significant role in controlling particle morphology (Tables S1 and S2). Ascorbic acid acts both as a reducing agent and a morphology-directing factor, influencing the nucleation and growth rates. High ascorbic acid relative to CuCl_2_ promotes anisotropic structures, such as chain-like nanostructures, whereas low ascorbic acid with high CuCl_2_ leads to larger, irregular particles. Indeed, comparative analysis showed that a slight excess of ascorbic acid ensures the complete reduction of Cu^2+^ ions to Cu^+^, promoting the selective formation of Cu_2_O with a regular spherical shape, high crystallinity, and monodispersity, as confirmed by TEM (Figure S1). Furthermore, XRD analysis performed after 3 months of storage at room temperature confirmed the absence of peaks corresponding to CuO. This result confirms that the nanoparticles maintain their phase purity and structural stability under the tested storage conditions (Figure S2).

The time interval between the addition of NaOH and ascorbic acid also proved to be a key factor in controlling the nanoparticle morphology. A short interval (<5 min) resulted in uncontrolled reduction and particle aggregation, whereas a prolonged interval (>5 min) led to premature reduction, producing heterogeneous sizes and poor control over the crystal phase. An optimized interval of 5 min enabled the controlled nucleation and growth of monodisperse Cu_2_O nanoparticles (Table S3 and Figure S3).

The pH of the reaction mixture critically influences nanoparticle formation. Higher volumes of NaOH solution (0.2 M, 3.0–3.5 mL) lowered the pH below 7.0, creating acidic conditions that compromised Cu_2_O stability. In contrast, using 2.0–2.5 mL of NaOH maintained the pH around 7.4, providing an optimal alkaline environment that ensured controlled nucleation and uniform particle formation, which, in turn, influenced the efficiency of nanoparticle internalization in fibroblasts (Table S4).

Previous studies have reported the biological effects of Cu_2_O and CuO nanoparticles from both green synthesis routes and commercial nanoparticles, showing comparable cytotoxicity trends in fibroblasts and tumor cells. Nonetheless, these nanoparticles often suffer from limitations, such as broad size distribution, poor monodispersity, or irregular morphologies. Ansarifard et al. demonstrated the biocompatibility of green-synthesized CuO nanoparticles in fibroblasts, but the particles were heterogeneous in size and shape.^38^ Hassabo et al. explored the anticancer properties of Cu_2_O nanoparticles, highlighting their potential as therapeutic agents against various cancer cell lines.^33^ On the other hand, commercial CuO nanoparticles, studied by Shafagh et al., display selective cytotoxicity toward the K562 cell line, inducing apoptosis without affecting healthy cells but generally lack uniformity and batch-to-batch reproducibility.^39^

Our optimized protocol produced monodispersed, spherical Cu_2_O nanoparticles with a narrow size distribution, achieved by fine-tuning the ascorbic acid/CuCl_2_ molar ratio, reaction time, and pH. Structural uniformity ensures reproducibility and reliable correlation between nanoparticle physicochemical properties and biological outcomes. Thus, while the cytotoxic profiles are consistent with previous reports, superior control over nanoparticle morphology represents a significant advancement, providing a robust platform for biomedical applications.

TEM, XRD, and DLS results show that the Cu_2_O NPs synthesized by this method have a highly crystalline nature and excellent dispersity, along with colloidal phase stability over time. These findings suggest that the synthesis and storage conditions are effective in preventing aggregation or significant variations in the size distribution.

The Cu_2_O nanoparticles exhibit a concentration-dependent antioxidant profile, as demonstrated by both the DPPH radical scavenging assay and their peroxidase-like catalytic activity. The DPPH assay revealed a dose-dependent biphasic behavior. At low concentrations (1–10 μg/mL), they exhibited significant antioxidant activity (IC_50_ = 7.05 μg/mL), whereas higher concentrations led to decreased scavenging efficiency, reflecting a transition toward pro-oxidant behavior. This pattern is consistent with the redox cycling of Cu^+^/Cu^2+^, capable of generating reactive oxygen species at elevated doses, and was confirmed by the intracellular oxidative stress assessment in 3T3 cells after 24 h exposure to Cu_2_O NP using the CM-H_2_DCFDA probe. FTIR analysis evidence the lack of residual ascorbic acid, demonstrating that the observed antioxidant activity is intrinsic to the nanoparticles. The peroxidase-like activity of Cu_2_O nanoparticles was evaluated across a concentration range of 0.01–0.09 mM, which corresponds to 1.4–15 μg/mL, directly overlapping with the low concentration values tested in the DPPH radical scavenging assay. These results indicate that Cu_2_O NPs can efficiently decompose hydrogen peroxide and mimic peroxidase activity within the same concentration range where they exhibit strong antioxidant activity in the DPPH assay (1–10 μg/mL).

Comparing Alamar Blue assay results and CM-H_2_DCFDA data with the DPPH radical scavenging assay, a strong agreement can be observed. Overall, the data reveal a dose-dependent dual behavior of Cu_2_O nanoparticles. At low concentrations, the nanoparticles exhibited significant antioxidant activity (IC_50_ = 7.05 μg/mL) in the DPPH assay while maintaining high cell viability (>90%), normal fibroblast morphology, and no oxidative stress, confirming their biocompatibility within the 0.07–7.15 μg/mL range. At higher concentrations, a progressive shift from antioxidant to pro-oxidant activity was observed in the DPPH assay, consistent with the redox cycling of copper ions (Cu^+^/Cu^2+^), which can trigger ROS generation and induce oxidative stress, as confirmed with the CM-H2DCFDA assay. The transition in the Cu_2_O NP behavior parallels the marked decline in 3T3 cell viability (IC_50_ = 16.16 μg/mL) and the appearance of morphological alterations, providing direct mechanistic support for the hypothesis that the onset of cytotoxicity is driven by the failure of the antioxidant phase and the activation of the pro-oxidant phase. Oxidative stress is a well-established central mechanism of cytotoxicity across diverse systems and cell types,^40,41^ and based on the obtained results, it represents the underlying mechanism accounting for the cellular cytotoxicity observed in 3T3 cells at higher Cu_2_O concentrations. Together, these results demonstrate that the redox-dependent biphasic activity of Cu_2_O nanoparticles translates into biological outcomes, underscoring the importance of concentration in determining their potential applications within a safe concentration range. Although the data support a concentration-dependent transition of Cu_2_O nanoparticles from antioxidant to pro-oxidant behavior, the possible contribution of released copper ions to ROS generation and cytotoxicity cannot be excluded. Future work should include copper release measurements under biologically relevant conditions to better define the possible role of Cu^+^/Cu^2+^ cycling and distinguish nanoparticle-specific effects from ion-mediated mechanisms.

Notably, the consistency between the IC_50_ value for DPPH radical scavenging (7.05 μg/mL) and the identified non-cytotoxic threshold (∼7.15 μg/mL) suggests a potential range of in vitro biocompatibility for Cu_2_O nanoparticle biomedical application. Therefore, for biomedical translation, maintaining dosages within this subthreshold range is essential to exploit their protective effects while avoiding the pro-oxidant-driven damage associated with higher copper loads.

Kinetic analysis, performed in the concentration range 1.4–12.6 μg/mL, revealed the catalytic efficiency of Cu_2_O nanoparticles in oxidative reactions and high substrate affinity. Their ability to catalyze reactions involving hydrogen peroxide H_2_O_2_ and 3,3′,5,5′-tetramethylbenzidine (TMB) suggests that Cu_2_O NPs participate in redox processes. Obtained results indicate that Cu_2_O NPs can efficiently decompose hydrogen peroxide and mimic peroxidase activity within the same concentration range where they exhibit strong antioxidant activity in the DPPH assay (1–10 μg/mL) and maintain high fibroblast viability (>90%). This concordance suggests that at low concentrations, the intrinsic peroxidase-like catalytic activity may contribute to the mitigation of potential oxidative stress conditions. Such activity could occur in the extracellular environment or within the cellular microenvironment, potentially complementing direct radical scavenging mechanisms. Taken together within the experimental conditions and the specific cell line tested, the integration of DPPH assay, kinetic analysis, cytotoxicity, and CM-H_2_DCFDA data allows the identification in vitro of a “safe” antioxidant concentration range for Cu_2_O nanoparticles, in which their intrinsic radical scavenging and catalytic properties provide effective oxidative stress mitigation without compromising cellular health.

Observed properties of Cu_2_O nanoparticles highlight their potential relevance in different fields, from biomedical to cosmetic and environmental, although further investigations are required to assess their applicability. In the biomedical context, the antioxidant and catalytic activities demonstrated in vitro suggest a possible role in strategies aimed at mitigating oxidative stress, but in vivo studies are essential before considering any therapeutic use. Regarding cosmetic applications, Cu_2_O nanoparticles could be incorporated into products designed to protect skin from oxidative damage caused by UV radiation, pollution, and other environmental stressors. In the environmental field, Cu_2_O NPs could be useful as catalysts to break down hazardous organic compounds in wastewater by mimicking the natural peroxidase enzyme, which helps in the degradation of pollutants such as phenolic compounds, industrial dyes, and pesticides.

## 5 Conclusions

Monodispersed Cu_2_O nanoparticles with a spherical shape were produced by a simple and low-cost chemical synthesis method. The biocompatibility of Cu_2_O NPs, demonstrated by their nontoxic effect on murine fibroblasts at concentrations near their DPPH scavenging EC_50_ value, highlights their potential biomedical applications. The polydispersity index (PDI) remained below 0.2 even after six months at room temperature, confirming the long-term stability of the nanoparticle dispersion. Colloidal stability and cellular uptake is crucial in ensuring minimal toxicity of the prepared Cu_2_O NPs. The values obtained from antioxidant activity revealed that while ascorbic acid provides superior antioxidant activity, its rapid degradation and short biological half-life may limit its sustained effect in therapeutic applications. In contrast, Cu_2_O NPs could offer a more stable and controlled antioxidant response over time. FTIR analysis confirmed the absence of ascorbic acid in the final formulation, suggesting that the antioxidant activity observed is an intrinsic property of the Cu_2_O NPs rather than any residual synthesis reagents. On the other hand, kinetic analysis of Cu_2_O NPs as peroxidase-mimicking nanozymes revealed a good catalytic efficiency in oxidative reactions. The ability to catalyze reactions involving hydrogen peroxide (H_2_O_2_) and 3,3′,5,5′-tetramethylbenzidine (TMB) suggests that Cu_2_O NPs participate in redox processes. These findings highlight that Cu_2_O nanoparticles possess intrinsic antioxidant properties, exhibit enzyme-like activity, maintain a stable physicochemical profile, have good biocompatibility, and show strong potential for biomedical applications. These promising results pave the way for future studies into the functional protective effects of Cu_2_O NPs in cellular models of oxidative stress, for example, by evaluating their ability to mitigate ROS generation and preserve cell viability in H_2_O_2_-challenged cells within the identified safe concentration range.

## Supplementary Material



## Notes

Stoichiometry with low concentration of AA and various concentrations of CuCl_2_ tested with the first batch (Table S1) and second batch (Table S2). Time interval used between the addition of NaOH and AA (Table S3). Different amounts of NaOH added to the mixture of CuCl_2_ and PVP before the addition of AA (Table S4). Concentrations of Cu_2_O nanoparticles used in the colorimetric assay shown in Figure 3 (Table S5). Comparative analysis of synthesis parameters, physicochemical characteristics, and biological activity (IC_50_) of Cu_2_O nanoparticles reported in this study and selected literature (Table S6). Figure S1. Chain-like NPs obtained from the molar ratio of 3:0. NPs with larger size and irregular shape obtained from the molar ratio of 1:6. Highly monodispersed with a quasi-spherical shape obtained in optimal conditions by using a molar ratio of 2:1. Figure S2. XRD pattern of nanoparticles recorded after 3 months of storage at room temperature to evaluate their long-term stability. Figure S3. NPs obtained after a long time interval reaction between NaOH and AA (10 min). NPs obtained after a premature reaction time (1 min). Figure S4. Color change observed in the colorimetric assay: TMB + H_2_O_2_ (no nanoparticles)—colorless; TMB + H_2_O_2_ + Cu_2_O nanoparticles—blue, due to the formation of oxidized TMB (oxTMB). Figure S5. Absorption spectra of DPPH (negative control) and Cu_2_O nanoparticles (NPs) in the absence of DPPH, recorded at the wavelength of 517 nm used for the scavenging assay. (PDF)
