# Supplementary material for: Antioxidant Properties and In Vitro Biocompatibility of Monodispersed Cuprous Oxide (Cu2O) Nanoparticles
Source: ACS Omega. 2026 Aug 6;11(32):47817–31. doi: 10.1021/acsomega.6c03242 (PMC13491954; doi:10.1021/acsomega.6c03242)
Supplement: Supplementary file 1 [file ao6c03242_si_001.pdf]

Supporting Information.

Antioxidant properties and *in-vitro* biocompatibility of monodispersed cuprous oxide (Cu_2O) nanoparticles

Laura Maria Slavu[1], Maria Elena Giordano[2], Sanosh Kunjalukkal Padmanabhan[3], Gayatri Udayan[2], Giorgio Capuzzelo[1], Antonio Licciulli[4] , Maria Giulia Lionetto[2] and Rosaria Rinaldi[1].*

[1]Department of Mathematics and Physics " Ennio De Giorgi" , University of Salento, Via Monteroni, Lecce 73100, Italy.

[2]Department of Biological and Environmental Sciences and Technologies (DiSTeBA), University of Salento, 73100 Lecce, Italy

[3] Department of Experimental Medicine, University of Salento, 73100 Lecce, Italy.

[4] Department of Engineering for Innovation, University of Salento, 73100 Lecce, Italy.

**sanosh.padmanabhan@unisalento.it*

Stoichiometry Effect

In the tables are summarized the amount of Ascorbic Acid and CuCl2 present in the reaction solution. Here two different experiments have been conducted. In both conditions the concentration of $CuCl_2$ has been variated while the concentration of AA has been maintained fixed firstly at 0.25 mmol and then at 0.3 mmol respectively.

Ascorbic Acid	CuCl2	Molar Ratio
0.25 mmol	0.01 mmol	2:5
0.25 mmol	0.015 mmol	1:6
0.25 mmol	0.2 mmol	1:25
0.25 mmol	0.25 mmol	1:1

Table S1 Stoichiometry with low concentration of AA and various concentrations of $CuCl_2$ tested with the first batch.

Ascorbic Acid	CuCl2	Molar Ratio
0.3 mmol	0.01 mmol	3:0
0.3 mmol	0.015 mmol	2:1
0.3 mmol	0.2 mmol	1:5
0.3 mmol	0.25 mmol	1:2

Table S2 Stoichiometry with higher concentration of AA and various concentrations of $CuCl_2$ tested with the second batch.

Ascorbic acid is well known for its dual role in the synthesis process, acting not only as a reducing agent but also as a morphology-directing factor. Its concentration significantly influences both the nucleation and growth rates, thereby modulating the size and shape of the resulting nanoparticles. Higher concentrations of ascorbic acid may promote anisotropic growth, leading to the formation of well-defined nanostructures, whereas lower concentrations typically result in spherical particles or aggregated forms.

In our study, it was observed that a high concentration of ascorbic acid combined with a lower concentration of $CuCl_2$ (molar ratio 3:0) favoured the formation of chain-like nanostructures. In contrast, lower concentrations of ascorbic acid and higher concentrations of $CuCl_2$ (molar ratio 1:6) led to the formation of larger nanoparticles with irregular shapes. Based on a comparative analysis of the various molar ratios tested, the optimal condition for synthesizing uniform and highly monodispersed Cu_2O nanoparticles was identified at a 2:1 molar ratio of ascorbic acid to $CuCl_2$. Some examples of nanoparticles obtained under different synthesis conditions are shown in the figure below.

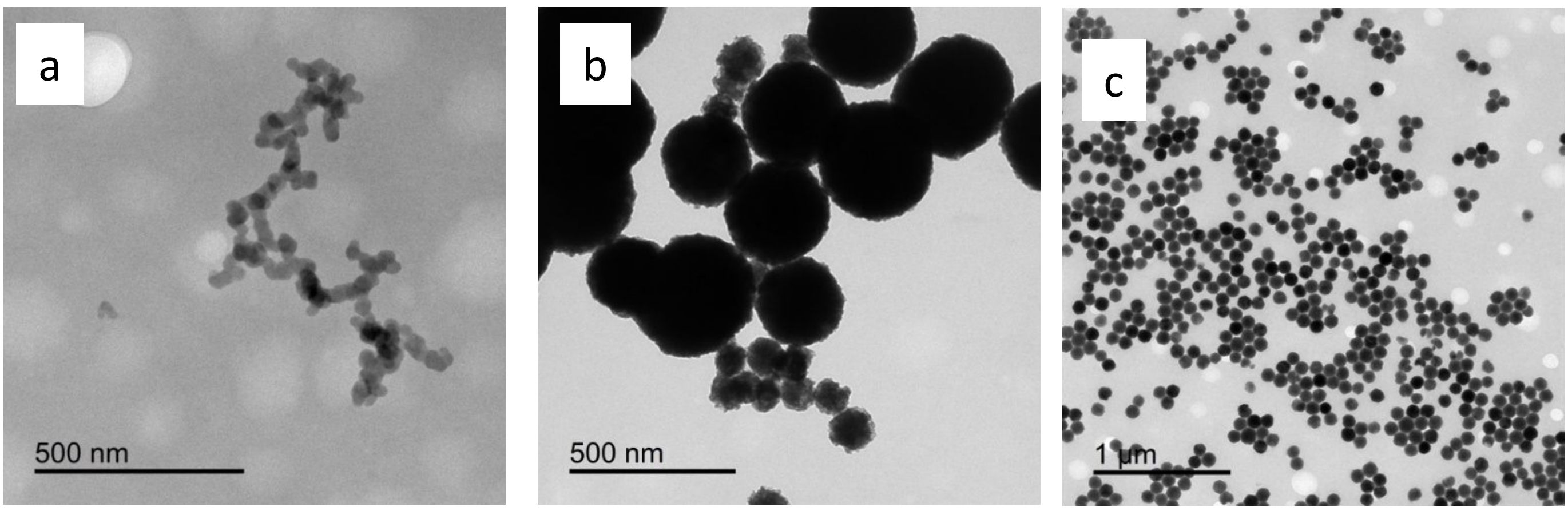

Figure S1 a) Chain-like NPs obtained from the molar ration of 3:0. b) NPs with larger size and irregular shape obtained from the molar ratio of 1:6. c) Highly monodispersed with a quasi-spherical shape obtained in optimal conditions by using a molar ratio of 2:1.

XRD Data Analysis.

The results show that the characteristic diffraction peaks of Cu_2O remain unchanged over three months at room temperature, with no detectable peaks corresponding to CuO. This confirms that the nanoparticles maintain their phase purity and structural stability under the tested storage conditions.

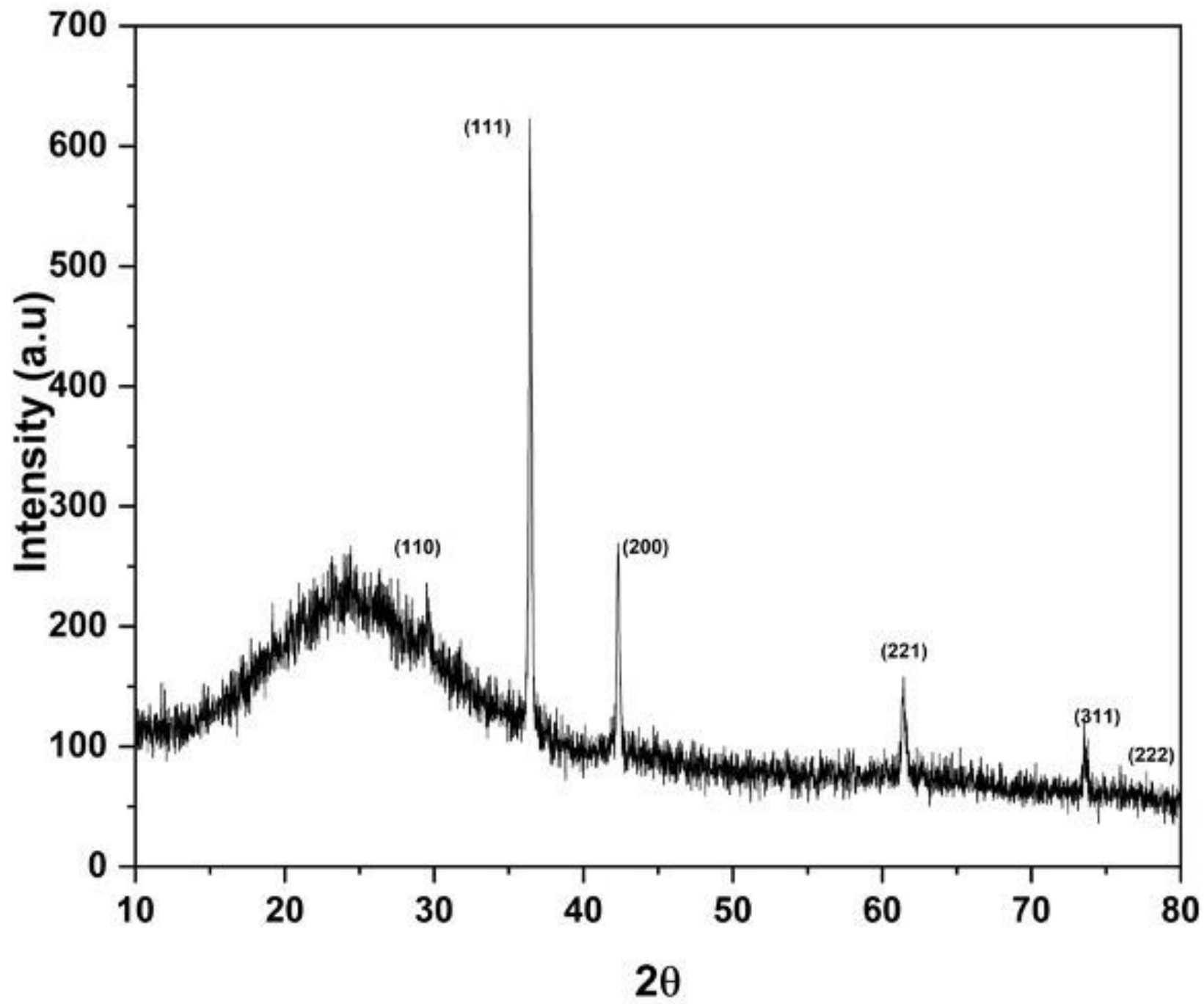

Figure S2. XRD pattern of nanoparticles recorded after 3 months of storage at room temperature to evaluate their long-term stability. The persistence of the characteristic cubic peaks and the complete absence of or metallic reflections confirm the high phase stability and resistance to oxidation of the synthesized nanoparticles.

Reaction time effect

To determine the optimal time interval between the addition of NaOH and ascorbic acid, different batches of nanoparticle solutions were prepared and tested under various conditions. The table below shows the reaction time interval between NaOH and AA and the polydispersity index (PDI) obtained from DLS analysis. From this data, it was observed that an optimal reaction time of 5 minutes results in a PDI greater than 2.0, which is considered excellent under these conditions. In contrast, shorter or longer intervals than 5 minutes resulted in non-specific or poorly defined nanoparticle morphology with higher PDI values, likely due to premature or delayed reduction processes that promote aggregation or uneven growth. These findings highlight the critical role of reaction timing in controlling nanoparticle size uniformity and quality. The figure below shows some examples of nanoparticles obtained after both long and short reaction times.

NaOH	Ascorbic Acid	Time Interval	PDI
2.0 mL	2.0 mL	1 min	0.316 ± 0.112
2.0 mL	2.0 mL	2.5 min	0.287 ± 0.127
2.0 mL	2.0 mL	5 min	0.107 ± 0.10
2.0 mL	2.0 mL	7.5 min	0.398 ± 0.147
2.0 mL	2.0 mL	10 min	0.594 ± 0.132

Table S3 – Time-Interval used between the addition of NaOH and AA

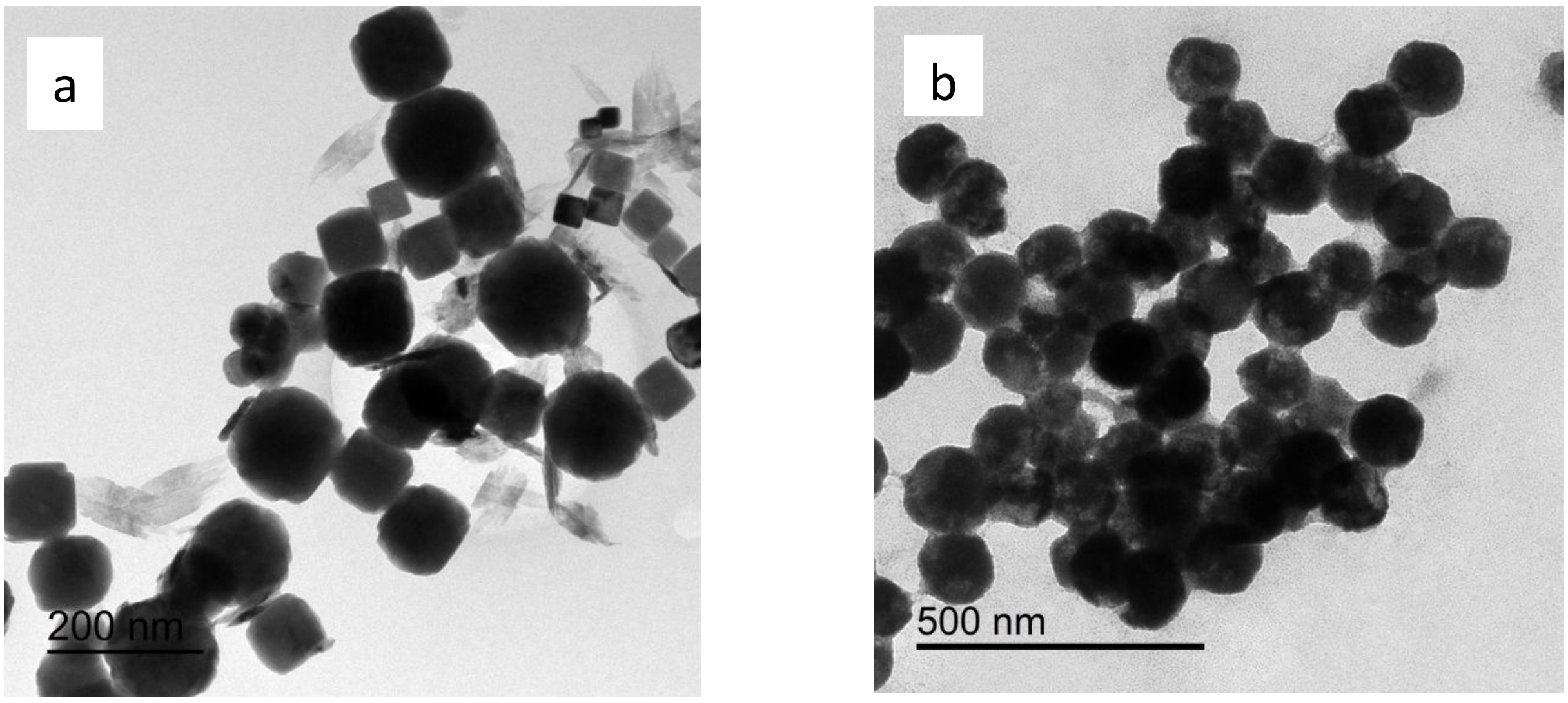

Figure S3. a) NPs obtained after a long time interval reaction between NaOH and AA (10 min). b) NPs obtained after a premature reaction time (1 min).

pH Effect

The pH was investigated across different batches of nanoparticles by adding various volumes of 0.2 M NaOH to a mixture of $CuCl_2$ and PVP (Table 3) in order to determine the optimal pH conditions for the nanoparticle solution. It was observed that when the volume of NaOH solution was increased to 3.0 or 3.5 mL, the pH of the reaction mixture dropped below 7.0, resulting in acidic conditions, which are not optimal. On the other hand, when 2.0 or 2.5 mL of NaOH was used, the pH of the mixture was around 7.4, providing an ideal alkaline environment for Cu_2O formation, allowing for a moderate reaction rate and controlled nucleation. Interestingly, the amount of NaOH added also significantly influenced the color evolution of the solution during synthesis. When 2.0 mL of 0.2 M NaOH was used, the solution underwent a characteristic color transition from blue to green to reddish-orange, indicating the progressive formation of Cu_2O nanoparticles. By increasing the NaOH volume to 3.0 or 3.5 mL, a more intense blue-to-green shift was observed initially, with a final cloudy orange color, most probably due to excessive hydroxide promoting the formation of $Cu(OH)_2$ or even CuO under certain conditions.

NaOH	NaOH Conc	pH
2.0 mL	0.2 M	7.4
2.5 mL	0.2 M	7.9
3.0 mL	0.2 M	9.2
3.5 mL	0.2 M	11.3

Table S4. Different amounts of NaOH added in the mixture of CuCl2 and PVP before the addition of AA.

Peroxidase-Mimicking Activity Assay Using Colorimetric Method

Peroxidase Colorimetric test for TMB (1 mM) and H2O2 (5 M)

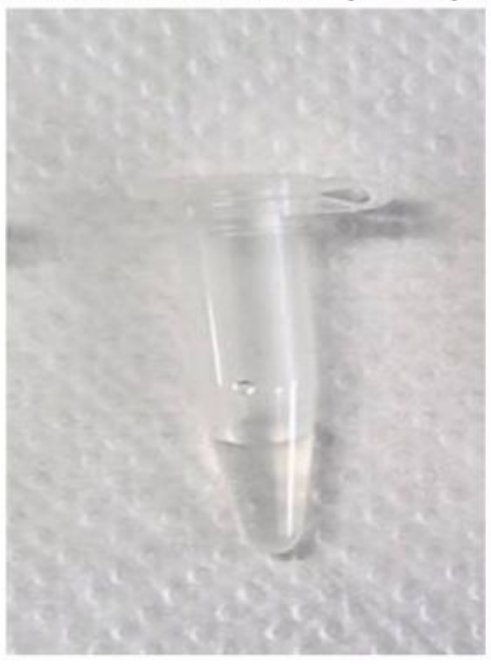

Peroxidase Colorimetric test after the addition of Cu2O NPs in the system at different concentrations

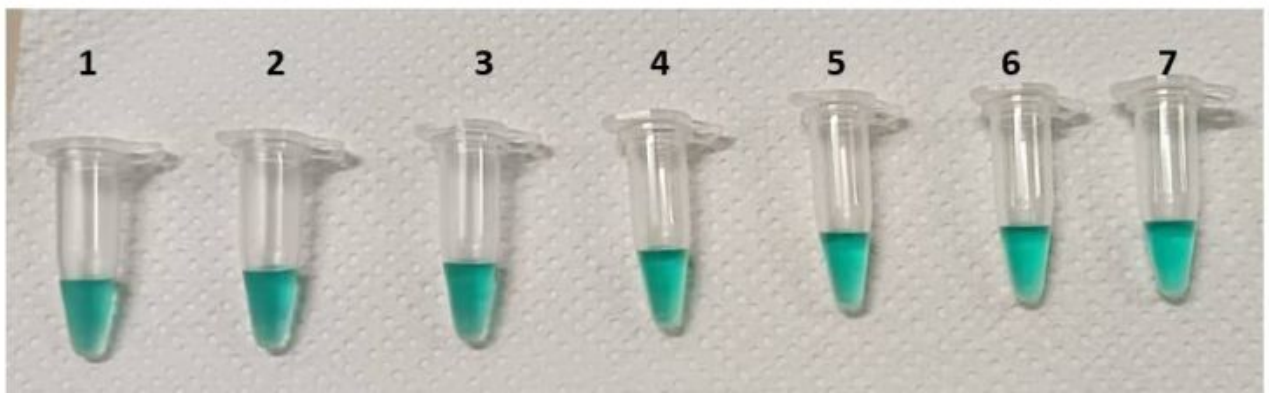

Figure S4: Colour change observed in the colorimetric assay: (top) TMB + H_2O_2 (no nanoparticles) – colourless; (bottom) TMB + H_2O_2 + Cu_2O nanoparticles – blue, due to the formation of oxidized TMB (oxTMB).

Name of the Sample	Conc of Cu_2O NPs	Conc of TMB	Conc of H_2O_2
1. Cu_2O	1×10^{-5} [M]	1 mM	5 [M]
2. Cu_2O	2×10^{-5} [M]	1 mM	5 [M]
3. Cu_2O	3×10^{-5}[M]	1 mM	5 [M]
4. Cu_2O	4×10^{-5} [M]	1 mM	5 [M]
5. Cu_2O	5×10^{-5} [M]	1 mM	5 [M]
6. Cu_2O	6×10^{-5} [M]	1 mM	5 [M]
7. Cu_2O	7×10^{-5} [M]	1 mM	5 [M]

Table S5 .Concentrations of Cu_2O nanoparticles used in the colorimetric assay shown in Figure 3. Each sample (1–7) corresponds to the labelling in the figure, representing increasing concentrations of Cu_2O nanozymes tested for peroxidase-like activity.

Study	Synthesis method	Reducing agent / Stabilizer	Size (nm)	PDI	Shape	Tested Cell line	IC50/Cytotoxicity	Reference
**This work**	**Chemical reduction with controlled stoichiometry, reaction time and pH**	**Ascorbic Acid/PVP**	**88 ± 6**	**0.107**	**Quasi spherical**	**3T3 Murine Fibroblast**	**Negligible at low conc. Pro-oxidant at high conc.**	
Cao et al., 2021.	Green synthesis, plant extract	Biomolecules/PVP	35-85	n.r	Mostly spherical. Some aggregation	Melanoma cells (MTT assay)	Cytotoxic at low-medium conc.	[1]
Umme Thahira. K et al. 2019	Chemical/green synthesis	$NaBH_4$ or plant extract	50-150	n.r	Irregular. Some aggregation	Human cancel cell lines (MTT assay)	Cytotoxic at low-medium conc.	[2]
Ansafrid et.al 2023	Hydrothermal synthesis	Glucose	120-200	n.r	Irrergular	Cancer cell line	Moderate cytotoxicity	[3]
Hassabo et al., 2022	Green synthesis	Plant extract/biomolecuels	50-150	n.r	Spherical	Fibroplasts	Moderate toxicity	[4]
Rajaram et al., 2023	Green chemical reduction	L-AA/HP-β cyclodextrin	33.2	n.r	Spherical	MDA-MB-231 breast cancer cells	~ 50% toxicity at 2mg/mL	[5]

Table S6. Comparative analysis of synthesis parameters, physicochemical characteristics, and biological activity (IC_{50}) of Cu_2O nanoparticles reported in this study and selected literature.

Antioxidant test

In the DPPH assay, the intrinsic optical absorbance of Cu_2O nanoparticles at 517 nm was taken into account, as it can overlap with the DPPH signal. For this reason, full control experiments (DPPH alone and nanoparticles alone) were systematically performed during the experimental work and are now reported in the Supporting Information, although they were not originally included due to an editorial omission.

The absorbance contribution of the nanoparticles alone was quantified over the concentration range 0.01–0.1 mM (1–100 µg/mL), showing relatively low values at 517 nm (Abs = 0.05–0.15) compared to the DPPH control (Abs = 0.903). These background signals were used as blanks and subtracted for each corresponding concentration in order to isolate the true radical scavenging contribution.

After correction, a marked decrease in absorbance was observed in the 1–10 µg/mL range (e.g., Abs = 0.328 at 10 µg/mL), indicating a significant scavenging activity. In the absence of antioxidant effects, the expected combined absorbance would approximate the sum of the individual contributions (≈1.05), whereas the experimentally observed values are substantially lower, confirming a genuine DPPH reduction with an inhibition up to 69.77%.

At higher concentrations (25–100 µg/mL), a reduction in apparent scavenging efficiency is observed, consistent with an increasing contribution of nanoparticle background absorbance. This behavior aligns with the biological assays (Alamar blue and ROS), where higher concentrations are associated with oxidative stress and cytotoxic effects, suggesting a concentration-dependent switch in the bioactivity profile.

Overall, the consistency between the corrected chemical assay and the biological response indicates that the observed trends are not artefacts of optical interference, but reflect the intrinsic concentration-dependent redox behavior of the nanoparticles.

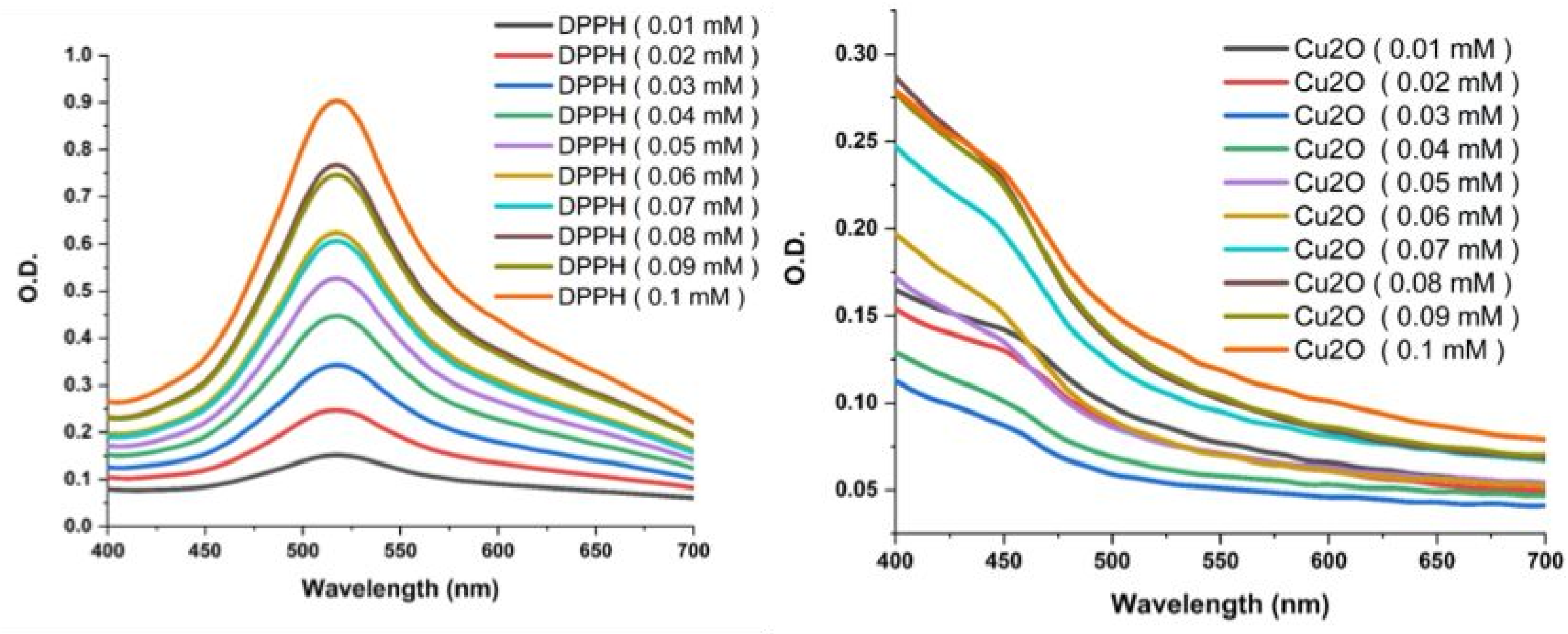

Figure S5: Absorption spectra of DPPH (negative control) and Cu_2O nanoparticles (NPs) in the absence of DPPH, recorded at the wavelength of 517 nm used for the scavenging assay. The nanoparticles exhibit intrinsic absorbance at this wavelength, which, however, is significantly lower than the DPPH signal. These controls were used for background correction in antioxidant activity measurements.

.
